# RNA-Seq, Bioinformatic Identification of Potential MicroRNA-like Small RNAs in the Edible Mushroom *Agaricus bisporus* and Experimental Approach for Their Validation

**DOI:** 10.3390/ijms23094923

**Published:** 2022-04-28

**Authors:** Francisco R. Marin, Alberto Dávalos, Dylan Kiltschewskij, Maria C. Crespo, Murray Cairns, Eduardo Andrés-León, Cristina Soler-Rivas

**Affiliations:** 1 Department of Production and Characterization of Novel Foods, Institute of Food Science Research—CIAL (UAM + CSIC), Universidad Autónoma de Madrid, 28049 Madrid, Spain; cristina.soler@uam.es; 2 Laboratory of Epigenetics of Lipid Metabolism, Madrid Institute for Advanced Studies (IMDEA)—Food, CEI UAM + CSIC, Pabellón Central del Antiguo Hospital de Cantoblanco, 28049 Madrid, Spain; alberto.davalos@imdea.org (A.D.); carmen.crespo@imdea.org (M.C.C.); 3 School of Biomedical Sciences and Pharmacy, Faculty of Health and Medicine, The University of Newcastle, Callaghan, NSW 2308, Australia; dylan.kiltschewskij@newcastle.edu.au (D.K.); murray.cairns@newcastle.edu.au (M.C.); 4 Bioinformatics Unit, Institute of Parasitology and Biomedicine “López Neyra”, Spanish National Research Council (CSIC), 18016 Granada, Spain; eduardo.andres@csic.es

**Keywords:** miRNAs, milRNAs, fungi, *Agaricus bisporus*, white button mushroom, RT-qPCR

## Abstract

Although genomes from many edible mushrooms are sequenced, studies on fungal micro RNAs (miRNAs) are scarce. Most of the bioinformatic tools are designed for plants or animals, but the processing and expression of fungal miRNAs share similarities and differences with both kingdoms. Moreover, since mushroom species such as *Agaricus bisporus* (*A. bisporus*, white button mushroom) are frequently consumed as food, controversial discussions are still evaluating whether their miRNAs might or might not be assimilated, perhaps within extracellular vesicles (i.e., exosomes). Therefore, the *A. bisporus* RNA-seq was studied in order to identify potential de novo miRNA-like small RNAs (milRNAs) that might allow their later detection in diet. Results pointed to 1 already known and 37 de novo milRNAs. Three milRNAs were selected for RT-qPCR experiments. Precursors and mature milRNAs were found in the edible parts (caps and stipes), validating the predictions carried out in silico. When their potential gene targets were investigated, results pointed that most were involved in primary and secondary metabolic regulation. However, when the human transcriptome is used as the target, the results suggest that they might interfere with important biological processes related with cancer, infection and neurodegenerative diseases.

## 1. Introduction

Small RNAs (sRNAs) are a ubiquitous class of non-coding RNAs with an average size of 20–30 nt that are involved in RNA silencing pathways, also known as post-transcriptional gene silencing (PTGS) in plants, quelling in fungi and RNAi (RNA interference) in animals [1]. Despite a heterogeneous group of minor and less studied sRNAs such as tiny non-coding RNAs (tncRNAs), trans-acting siRNAs (tasiRNAs), etc. [2,3], microRNAs (miRNAs) are, together with short interfering RNAs (siRNAs) and PIWI-interacting RNAs (piRNA), the three major categories of sRNAs. piRNAs are associated with proteins from the Piwi class of animal Argonaute (AGO) proteins, do not require RNAse III for their maturation and play important roles in germline cells. siRNAs and miRNAs are associated with the AGO proteins and share important features, such as that both are produced by the Dicer ribonuclease and perform their biochemical functions mainly in somatic cell lines [1,4]. However, they also show some differences. For example, miRNAs are involved in the regulation of protein-coding genes through translational repression and messenger RNA (mRNAs) degradation while siRNAs are involved in antiviral defense. Moreover, miRNAs originate from precursors with a typical hairpin structure generating a single miRNAs (guide):miRNAs * (passenger) duplex, while siRNAs are processed from long bimolecular RNA duplexes, and a multitude of siRNA duplexes are generated from each siRNA precursor molecule [1,4].

miRNAs are considered the hallmark of the RNA silencing pathways to guide the selective degradation of mRNAs by cleavage, translational repression or transcriptional suppression of targets (mRNAs). These molecules were noticed in a wide variety of organisms, from large DNA viruses (Epstein–Barr and herpes viruses) [5] to amoebas, brown algae, nematodes, mollusks, tunicates, sea lampreys, insects, monocots, dicots, vertebrates and also in fungi [6] and in eukaryotes. Currently, miRNA studies mainly focus on animals or plants, leaving those in the fungal kingdom behind. Animal miRNAs are typically 22 nt in length and generated from miRNA-encoding genes that generate single-stranded RNA precursors with characteristic hairpin structures. They need specific proteins such as Drosha, Dicer, Argonaute, etc., together with RNA pol II, for their biosynthesis and export out of the nucleus [4]. Plant miRNAs are typically 21 nt in length and include a 2′-*O*-methylation at the 3′ end, showing a slightly different biosynthetic pathway than animals involving other enzymes and maturing steps until they are released into the cytoplasm [7]. In both cases, canonical and non-canonical biosynthetic pathways coexist [8]. For instance, in metazoan, non-canonical pathways produce only 1% of the reported miRNAs [4,9]. Although quelling was previously noticed [10], the presence of miRNAs in fungi was reported for the first time in *Neurospora* spp. as miRNA-like RNAs (milRNAs) [11]. Since then, several fungal milRNA candidates were identified. For this, deep sequencing technologies and bioinformatic tools are used, not only in other filamentous fungi [12,13,14,15,16,17,18], but also in basidiomycetes such as *Ganoderma* spp. [19,20] or *Antrodia cinnamomea* [21].

Experimental studies in *Neurospora* spp. indicated that fungal milRNAs share similarities and differences with miRNAs from other kingdoms. For instance, they share with their metazoan counterparts the origins from stem-loop RNA precursors, as observed for milR-1, the most abundant milRNA in *Neurospora crassa* (*N. crassa*) which requires Dicer, Ago and QIP proteins [6,11]. However, other milRNAs are generated by similar (but not identical) pathways to those of canonical miRNAs of plants and animals. Thus, four different biosynthetic pathways produce *N. crassa* milRNAs where different combinations of common elements participate together with others [22] and only one type of milRNAs is produced by a Dicer-independent pathway [11]. Moreover, their RNA precursors are transcribed mainly from intergenic regions, they show a strong preference for uracil at their 5′ end, and their average length is 25 nt for the mature form, although a wider range between 19 and 31 nt was reported [6].

On the other hand, there is an increasing amount of evidence on the relevance of miRNAs in the fields of Agronomy and Food Sciences. Thus, very recently the potential use of miRNAs as tools to increase pest tolerance in agronomic practices has been indicated [23,24]. In addition, the latest evidence shows that dietary plant miRNAs can not only be absorbed in the intestine, but also be absorbed and packaged by gastric epithelial cells and then secreted into the circulatory system. The former leads to consider miRNAs as biologically active and plant derived in a similar way as phytochemicals, and therefore, potentially responsible for food functionality [25].

Although *Agaricus bisporus* (the white button mushroom, *A. bisporus*) is one of the most consumed mushrooms worldwide and its genome was completely sequenced (H97 variety) [26], no information has been found so far describing the presence of sRNAs in its edible parts (fruiting body and stipe). Therefore, this work was aimed to identify putative milRNAs from *A. bisporus* using NGS (next-generation sequencing) and bioinformatic tools followed by an experimental approach with RTq-PCR to validate theoretical milRNA candidates. Furthermore, a prediction of potential targets on the *A. bisporus* genome, together with an approach to cross-kingdom putative regulation in humans was carried out, and a functional analysis of the regulated pathways both in mushroom and in human were also conducted. Finally, due to our theoretical predictions we expect to give a research framework for the experimental investigation.

## 2. Results

### 2.1. Analysis of Small RNA Library

**Figure 1:**
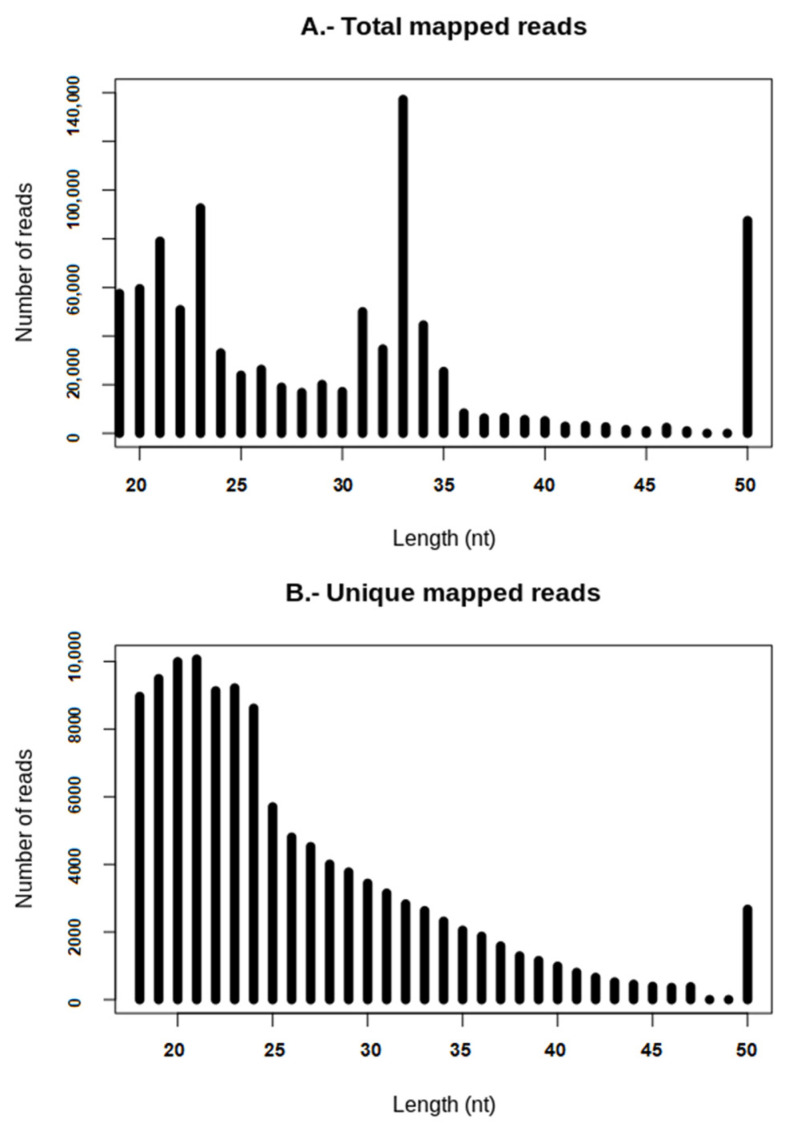
Length distribution of reads after mapping. (**A**) Total mapped reads. (**B**) Unique mapped reads.

A total of 2,027,870 raw reads were obtained from sequencing sRNAs from *A. bisporus* fruiting bodies (Table 1). After removing 3′ specific adapters, reads showed a Q_score_ > 32, so none were discarded. Quality analysis was also carried out after trimming with identical Q_score_ results. Thus, sequences with lengths lower than 18 nt were removed following the same criteria as previous studies carried out on other fungal species [16,19]; 1,421,021 (100%) total clean reads, corresponding to 291,880 (100%) unique reads, were obtained for further analysis. The clean reads were aligned against the *A. bisporus* genome assembly and only those with a perfect match were saved, resulting in 1,015,249 mapped reads (71.44%) where 117,838 of them were unique reads (40.37%). After mapping, the total and unique read length distribution in the range from 18 to 50 nt indicated that result. The most abundant lengths were 33 nt and 21 nt within the total and unique reads (Figure 1). In the range of sRNAs (20–30 nt), 18 nt was the most abundant length in total reads and 21 nt in unique reads.

To avoid artifacts in miRNA prediction, mapped reads were screened against ncRNAs from different databases (Table S1). The most restrictive results were obtained using Ensembl Fungi files with the exception of rRNA. For this group, NCBI files showed more matchings (25.56% of total mapped reads). Although this result may lead to consider that a high percentage of the reads originated from rRNA, it is necessary to highlight that during the RNA purification via PAGE no fragmentation was carried out, and therefore RNA molecules maintained their original size (plus adaptors <50 nt) and rRNA was not isolated except for degraded fragments. Thus, since the Ensembl Fungi file was particularly designed for *A. bisporus* and the obtained results were more consistent with the experimental protocol than the other files, it was selected for filtering (and typifying) the sRNA library (Table 1). Results indicated that a considerable number of raw reads were sourced from structural ncRNAs such as tRNA (26.45% total reads), while the regulatory ncRNAs (i.e., miRNAs, siRNAs, lncRNAs, etc.) were altogether only 11.93% of total reads. Within the unique reads, only a few were ncRNAs (6.08%), indicating that most of them showed other origins (93.92%). Thus, after filtering, the 626,577 total reads and 110,677 unique reads of unknown source were mapped to figure out their chromosomal and loci distribution.

**Figure 2:**
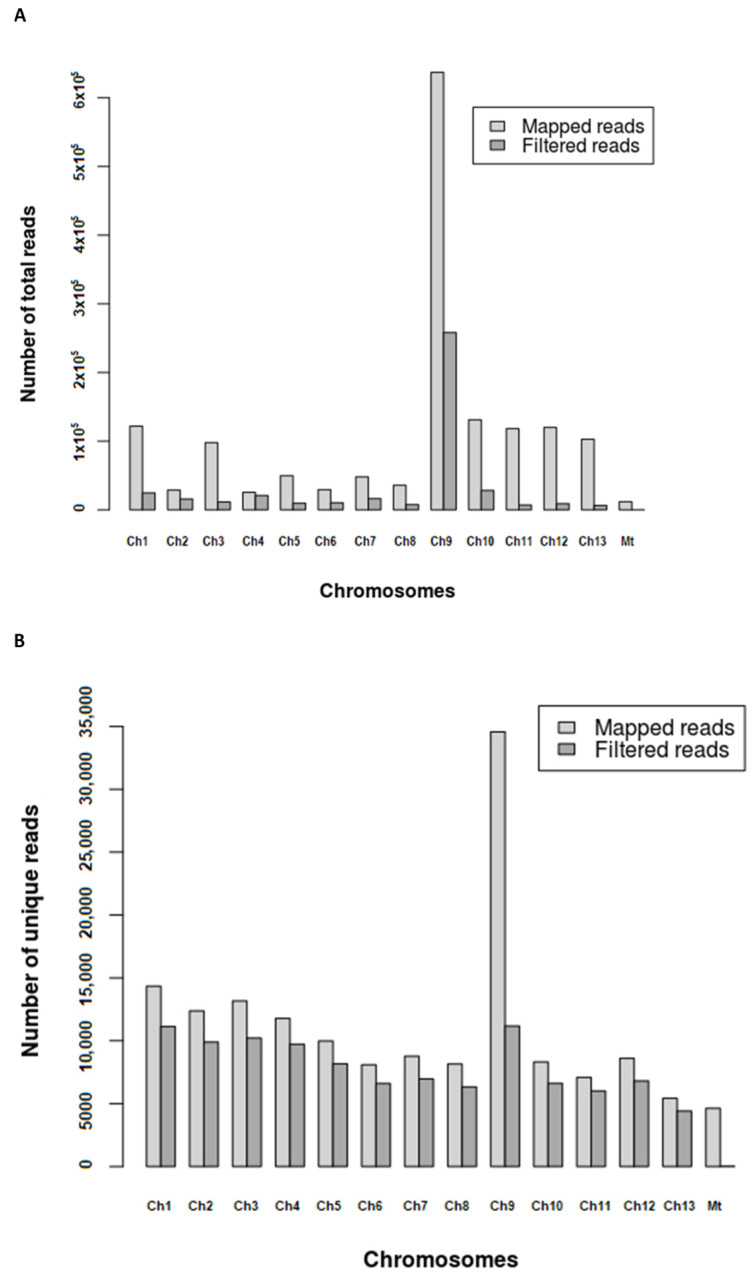
Read distribution on chromosomes (Ch) and mitochondrion (Mt) after mapping. (**A**) Distribution of total reads before and after removing ncRNAs. (**B**) Distribution of unique reads before and after removing ncRNAs.

**Figure 3:**
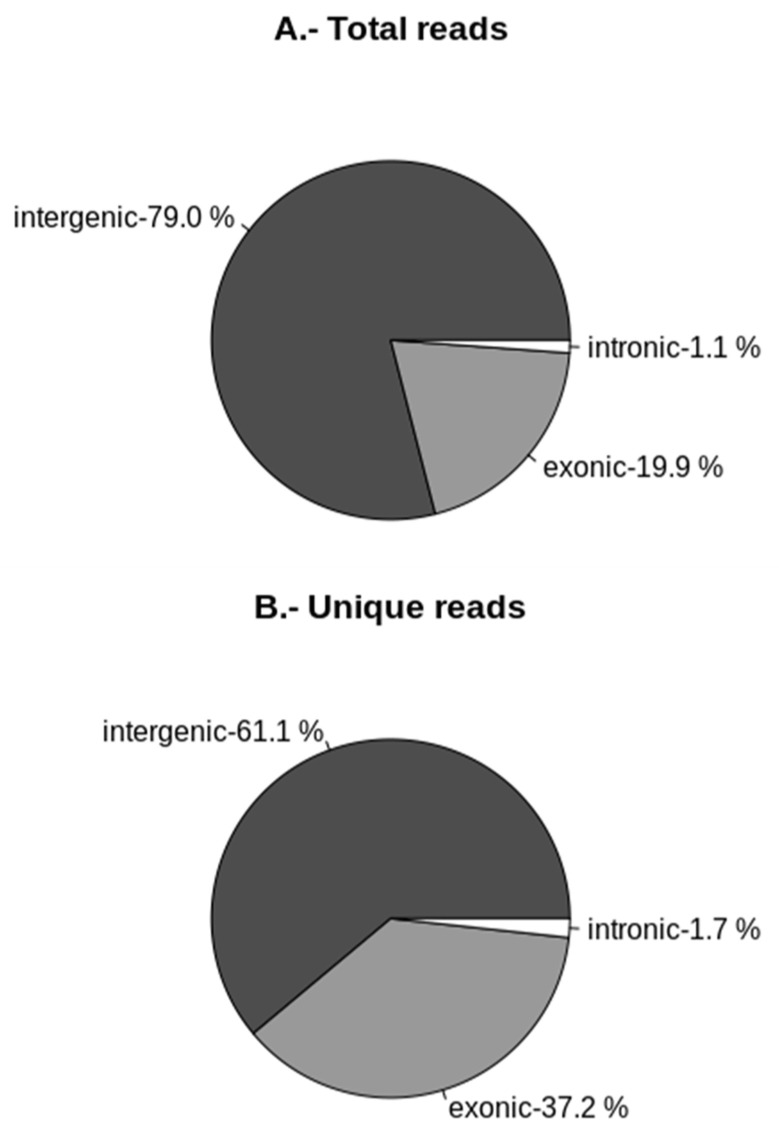
Small RNA loci annotation. Pie graphs show percentage of intergenic, exonic and intronic annotated reads. (**A**) Total reads. (**B**) Unique reads.

Most of the short RNA fragments (in the range 18–50 nt), before removing the ncRNAs, mapped on chromosome 9 (45% of all reads) (Figure 2A). This contribution fell to 22% when unique reads were mapped by chromosome (Figure 2B) and to 13% when reads were filtered (ncRNAs removed), and only reads in the range 18–30 nt (sRNAs) were mapped. Considering the relatively short size of the mitochondrial genome (approx. 135 kbp) [27] and the large number of short RNA fragments mapped (11,838 reads), mitochondrion could be pointed as an important source of sRNAs. However, after removing ncRNAs and mapping only reads in the range 18–30 nt (sRNAs), an exiguous number of 30 unique reads were found to originate from the mitochondrion. Moreover, the unknown reads obtained after filtering were also mapped to obtain their loci annotation, and results indicated that the intergenic regions were the main source of both total and unique reads, representing 79.0% and 61.1% of them, respectively (Figure 3). Exonic regions accounted for only 19.9% and 37.2% of total and unique reads, respectively, while the contribution of intronic regions was limited to a 1.1% for total and 1.7% for unique reads.

### 2.2. Homology Search among Known miRNAs

An attempt to find homologies using miARma-Seq was performed and resulted as unsuccessful. Therefore, a search against miRBase (release 21.0) was also carried out using BLAST and only three potential homologs were found among all reads. One of them matched with 67 miRNAs from different animal species and it belonged to the let-7 family, but it showed only two reads and was thus disregarded. The other one showed homology with the plant miRNA ptc-miR6478 and was present in higher number of reads (2060). The third potential homolog was discarded because the match was incomplete.

### 2.3. Prediction of De Novo milRNAs

**Figure 4:**
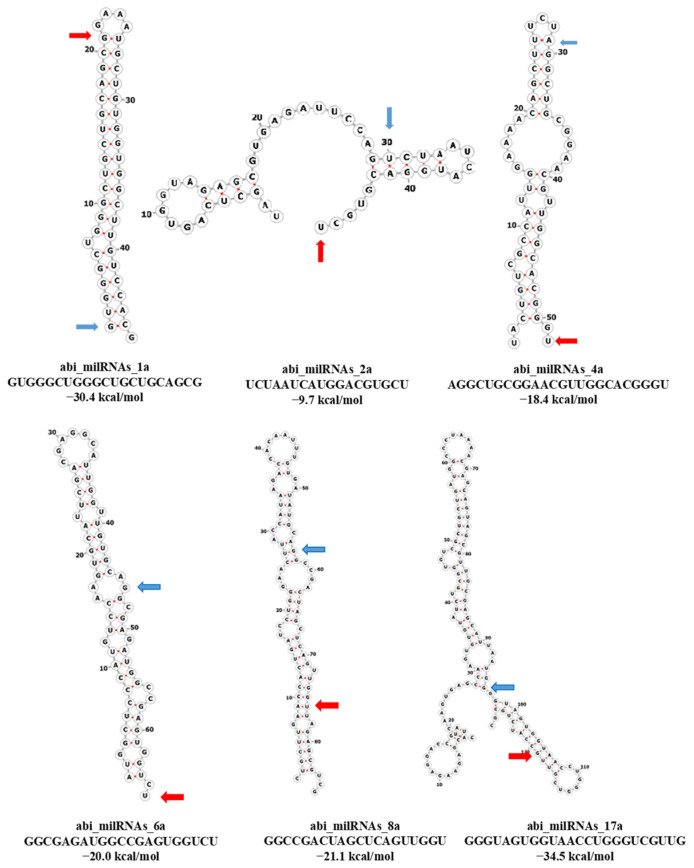
Secondary structures proposed for abi_milRNA precursors (abi_milRNA_1a, 2a, 4a and 6a are representative of those predicted by animal criteria and 8a and 17a of those with high homology with other fungi). The corresponding mature milRNA sequence and the estimated free energy of the pre-miRNAs are also indicated. Blue arrow: start mature sequence. Red arrow: end mature sequence.

Currently, no software is available to predict fungal milRNAs, perhaps because of the scarce knowledge about them. Therefore, software designed to predict both animal and plant miRNAs was used to look for potential milRNAs in *A. bisporus*. milRNA predictions following animal standards (with miRDeep2 and miARma-Seq) found six milRNA candidates with lengths ranging from 18 to 24 nt and a mode of 21 nt, and the secondary structures of their precursors could be drawn by RNAfold for abi_milRNA_1a, 2a, 4a and 6a (Figure 4), reinforcing the theoretical prediction as contiguous sequences can generate the precursor hairpin structure [1]. However, predictions following plant standards (with miRPlant) pointed to a considerably higher amount of 31 milRNA candidates, showing the same length range as miRDeep2 prediction but a mode of 22 nt. No candidates coexisted in the two predicted groups, and therefore all the potential milRNAs were listed one after the other (Table 2) and named using the nomenclature indicated in Griffiths-Jones, Grocock, Van Dongen, Bateman and Enright [28] and Desvignes et al. [29]. A double check was performed by using the milRNApredictor tool [30], some of them being (i.e., milRNAs abi_milRNA_1a, abi_milRNA_2a, abi_milRNA_4a, abi_milRNA_5a, abi_milRNA_6a, abi_milRNA_7a, abi_milRNA_9a, abi_milRNA_9b, abi_milRNA_11a, abi_milRNA_13a, abi_milRNA_16a, abi_milRNA_19a, abi_milRNA_20a, abi_milRNA_25a, abi_milRNA_26a, abi_milRNA_28a, abi_milRNA_30a, abi_milRNA_31a, abi_milRNA_32a, abi_milRNA_35a, abi_milRNA_36a) classified as fungal ones. However, the low training data used (<1300) should be noticed in evaluating the reliability of this last result.

**Figure 5:**
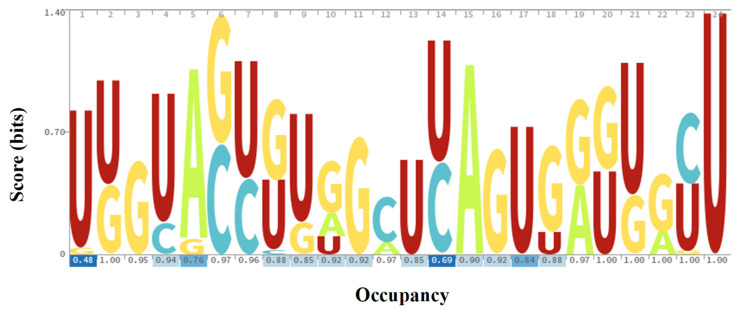
Sequence logo of predicted *A. bisporus* milRNAs. Logo represents score of weighted counts from a multiple alignment.

The sequence logo for the predicted milRNAs suggested a tendency to include more uracil and guanine in several positions than adenine or cytosine (Figure 5). Particularly, uracil was frequent in the 5′ and 3′ extremes. The prevalence to include uracil at the 5′ extreme was previously reported for fungi from other taxa [11,14,15,16] but also for the basidiomycetes such as *Ganoderma lucidum* [19]. Pre-milRNA sequences can be found as Supplementary Material in Table S2. Most of the pre-milRNAs are identical in length and sequence independent of their position (i.e., pre-milRNAs 12a, 18a and 20a), but not a couple of them (9a or 13a), which leads to carefully considering them as putative milRNAs.

### 2.4. Experimental Verification by RT-qPCR

**Figure 6:**
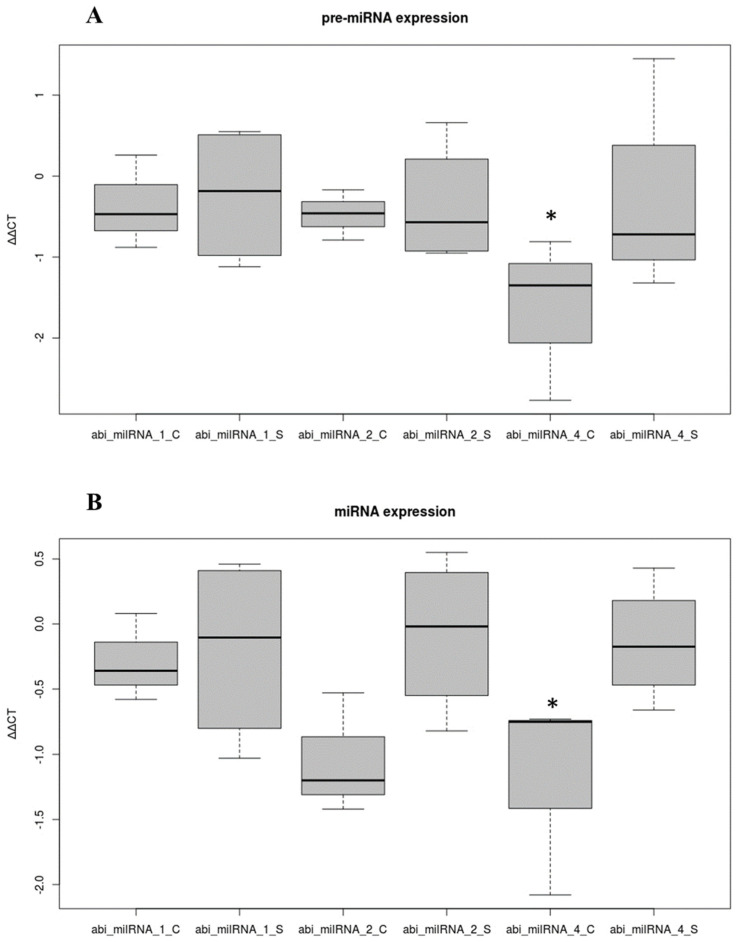
Verification of pre−miRNAs and miRNAs by RT−qPCR. (**A**) Differential expression of abi_milRNA_1a, abi_milRNA_2a and abi_milRNA_4a pre−miRNAs in stipe (S) and cap (C). (**B**) Differential expression of abi_milRNA_1a, abi_milRNA_2a and abi_milRNA_4a mature miRNAs in stipe (S) and cap (C). *: Results are statistically significant at α: 0.1.

Three of the predicted de novo milRNAs (abi_milRNAs_1a, abi_milRNAs_2a and abi_milRNAs_4a), following animal criteria, were selected for experimental verification using RT-qPCR attending to their abundance. The melting curves obtained from both cap and stipe samples showed a single peak of a pure and single amplicon, indicating that the designed primers achieved a proper specificity. The three predicted milRNAs were detected in both mature and precursor forms (CMS and CPS, respectively), suggesting their presence in the two mushroom tissues. The amplification curves showed CT values ranging from 8.4 to 9.7 for the qPCR to detect the mature and precursor forms of abi_milRNA_1a, from 21.7 to 22.0 for abi_milRNA_2a, and 33.0 to 31.0 for abi_milRNA_4a, while negative controls showed 39.0, 37.0 and 38.5, respectively. No statistically significant differential expression of pre-milRNAs was noticed between the tissues (Figure 6A). Moreover, for milRNA expression (Figure 6B), the ANOVA test did not show significant differences at α = 0.05 but it did at α = 0.1. A pair-wise Bonferroni test revealed differential expression for the abi_milRNA_4a within the cap and the stipe.

### 2.5. Homology with Other Fungal Species

The predicted milRNAs were mapped against a collection of representative Basidiomycetes genomes (Table S3), obtained from Ensemble Fungi (ftp://ftp.ensemblgenomes.org/pub/fungi/release-37/fasta/fungi_basidiomycota1_collection/, accessed on 14 May 2018). For a conservative approach, parameters such as only 0–1 mismatch and no length variation were selected. From those, only 19 milRNAs matched with any of the studied genomes. All of them except for one (abi_milRNA_6a) were predicted by miRPlant following biogenetic criteria for plants. The most ubiquitous *A. bisporus* de novo milRNAs were abi_milRNA_23a, abi_milRNA_17a, abi_milRNA_18a and abi_milRNA_8a, since they were found in 59, 55, 52 and 52 different species, respectively, and followed by abi_milRNA_6a (found in 49 species), predicted by miRDeep2. The predicted milRNAs perfectly matched with sequences detected in only 67 species, but they showed possible homology with 94 of them when one mismatch was allowed. *Leucoagaricus* spp., a species belonging to the same family as *A. bisporus* (*F. Agaricaceae*), showed the largest number of potentially identical homologues (a total of 13), followed by *Galerina marginata* and *Hebeloma cylindrosporum*, with 10 identical homologues. The former mushrooms are classified in a different family (*F. Strophariaceae*), but they are included, together with *A. bisporus*, into the *Agaricales* order. In fact, the species showing eight or nine homologues were mostly classified in the same taxonomic order (*Agaricales*) or in the close *Polyporales* order. Only one species, i.e., *Coniophora puteana*, showed a similar number of eight putative homologues and does not belong to *Agaricales* or *Polyporales* orders but to the *Boletales* order (all belonging to the Class *Agaricomycetes*). When the species were included in a different taxonomical class, the number of potential homologues fell down to one or zero. On the other hand, ptc-miR6478 did not match with other fungal species.

Moreover, a file including the sequences of fungal milRNAs described in the literature (up to 177 milRNAs) was created, highlighting those with experimental evidence, and a multiple aligning was carried out with T-Coffe to generate a preliminary phylogenetic correlation. Results indicated that several milRNAs shared internal nodes with those milRNAs with experimental evidence (Table 3). However, since the pointed species were included in a different division (*Ascomycota*) than *A. bisporus* (*Basidiomycota*), results could only suggest that the fungal milRNAs might have a common biogenesis.

### 2.6. Target Gene Prediction and Functional Analysis for A. bisporus milRNAs

*A. bisporus* milRNA targets were studied with a theoretical approach by using psRNATarget V2 on its reference genome and on the human transcriptome. Thus, both the 37 de novo predicted milRNAs and ptc-miR6478 were submitted to this tool, selecting default conditions. All the submitted milRNAs showed potential targets on the *A. bisporus* genome, with the exception of abi_milRNA_2a, abi_milRNA_25a, abi_milRNA_27a and ptc-miR6478. However, when a different software for target prediction was used, e.g., miRanda [31], under minimal restrictions (i.e., seed size and Gibbs free energy difference), targets for abi_milRNA_2a were also found.

A total of 6946 putative targets for 35 milRNAs were predicted, with cleavage (5727 times) being the most common mechanism versus translational repression (1219 times), which represents approximately 82% vs. 18%. A similar pattern was followed by each individual milRNA. In addition to this, 10,444 genes are listed in the KEGG database [32] for *A. bisporus* H97. From these, 4437 elements are regulated by the proposed milRNAs. Most of them correspond with proteins (4389), while a few correspond to tRNAs. After that, a functional analysis to identify the most regulated pathways was performed with the KEGG-Mapper tool [33]. According to the results, 109 pathways might be regulated by the proposed milRNAs, the top pathways being the basic metabolic pathways, with 304 nodes, and the biosynthesis of secondary metabolites, with 124 regulated nodes. Table 4 shows the 10 top regulated pathways for *A. bisporus* and the human transcriptome by the proposed milRNAs. However, these results should be viewed under an annotation bias. Thus, from the list of 10,444 elements, 6723 do not have a (KEGG Orthology) identifier or a putative function assigned, i.e., we only know the function of approximately 35% of the genetic elements of *A. bisporus*.

On the other hand, *A. bisporus* is a frequently consumed edible mushroom [34]. Thus, because it is necessary to clarify whether *A. bisporus* milRNAs might be absorbed with diet and reach their target in humans, we performed a theoretical approach on the human transcriptome. To perform this, we used two different miRNA target prediction tools, psRNATarget v2 and miRanda v3. As the aim was to obtain the intersection of both methods (using more than one miRNA prediction method is advisable [35]), we used the same set of 3′-UTR sequences from the human genome (obtained from 70). In the case of psRNATarget we used the default values, and in the case of miRanda we used a score cutoff (-sc) of 120 and gap opening and gap extension (-go, -ge) of -9 and -4, respectively (as recommended by the group in which miRanda was developed [30]).

In total, we obtained 1730 identical targets (same start:end coordinates in the 3′-UTR) between both methods). Among these, we can highlight as described above for the *A. bisporus* genome, that abi_milRNA_2a, abi_milRNA_25a and abi_milRNA_27a did not show potential targets on the human transcriptome. In addition to this, 22,214 genes are listed in the KEGG database [32] for *Homo sapiens*. From these, 19,572 code for proteins and 2642 code for RNA elements. According to the results, 319 pathways might be regulated by the prosed *A. bisporius* milRNAs. From these, as shown in Table 4, human basic metabolic pathways, with 113 regulated nodes, and human pathways in cancer, with 64 regulated nodes, were the most regulated ones. Furthermore, it should be noticed that 28 pathways are also related to infection processes (e.g., herpes simplex virus infection, with 52 regulated nodes, and human papillomavirus infection, with 40 regulated nodes) and 7 to neurodegenerative processes.

## 3. Discussion

The combination of high-throughput sequencing technologies and bioinformatic processing facilitates the identification of small RNAs in any organism, providing quantitative information about their transcription and sequence. Given that it is expected that they are highly conserved within closely related species, and share similar biosynthetic pathways, computational tools can be used to study them, i.e., the secondary structure of their precursor or miRNA mature sequences, to find homologies, to screen the complete genome of multiple species, or even to suggest their potential target genes among other applications. However, several of these programs were designed taking into consideration the particularities of plant or animal sRNAs, and therefore the proposed predictions for organisms from other kingdoms might be carefully studied and experimental confirmations should always be required. Therefore, for the identification of fungal milRNAs from the white button mushroom, several tools were utilized and compared, and an experimental approach was also carried out for some predicted miRNAs.

The ratios between clean reads (total reads and clean reads vs. unique clean reads) obtained after sRNA sequencing were similar to those previously obtained for other fungi, and the most abundant ones were those of 21 nt, after mapping for unique reads [16,19]. Other common features with fungal milRNAs were their genomic positions, intergenic regions being the major source of sRNA production [6,16], or their strong preference for 5′ U [11,15], suggesting that they all followed a common evolutionary pathway. Furthermore, the production of sRNAs in *A. bisporus* seems to follow a hot-spot pattern as described for other fungi [16]. Thus, chromosome 9 is the source of 45% of total reads and 22% of unique sequences. However, certain differences were also found when compared with less evolved fungi, such as *F. oxysporum*, where reads were mostly located in chromosomes 2 and 4, but also in the mitochondrion [16], which was finally irrelevant in *A. bisporus*. This hot-spot pattern has only been described, as mentioned, in Fusarium oxysporum, and no relevant features regarding repetitive elements are described for *A. bisporus* Chr9 [36]. The low number of identified milRNAs (1 known and 37 predicted) also diverged from other basidiomycetes such as *G. lucidum*, where 166 potential milRNAs were found [19]. The latter discrepancy might be because different bioinformatic tools and settings were used for their identification. Indeed, miRDeep2 uses an algorithm based in a probabilistic model of animal miRNA biogenesis that scores patterns and frequencies of the RNA sequences, taking into account the suggested secondary structure (as pre-miRNAs) obtained with RNAfold from the Vienna RNA package. In comparison, miRPlant was similarly designed, but for plant miRNAs following biogenetic plant criteria [37,38]. Although neither of the tools were specifically designed for fungal milRNAs, putative candidates were postulated because apparently the biogenesis of fungal milRNAs shares similarities with both plant and animal kingdoms, as previously mentioned [6].

On the other hand, initially we approached the search of *A. bisporus* milRNAs with an evolutionary bias, which assumes that the animal and fungal lineages share a more recent common ancestor than either does with the plant, alveolate or stramenopile lineages [39]. Thus, a prediction under biogenetic animal criteria yielded only six de novo milRNAs, and experimental validation, through qPCR, was carried out for three of them with positive results. However, when an approach based in different biogenetic criteria was conducted, we found a considerably higher number of de novo milRNAs (31 milRNAs). Unfortunately, the shortage of funds did not allow us to validate them experimentally. However, the aforementioned features (i.e., length, hairpin precursor, thermodynamic viability (evaluated through psRNATarget), match with genome, homology with related species, etc.) point more to a consistent prediction than a false one, which should be validated in further studies. Additionally, it should be highlighted that only canonical milRNAs can be predicted by using this approach. Further molecular validation of the above-mentioned proposed milRNAs, together with studies on their biological function, will provide definitive proof of their miRNA-like structure and function. Regarding the quantification presented here, it is important to note that even when the melting curves obtained from both cap and stipe samples showed a single peak of a pure and single amplicon—indicating that the designed primers achieved a proper specificity—the method used for analysis could eventually also quantify pri- and pre-miRNA sequences. Thus, the real level of expression should be considered with caution.

The higher number of fungal milRNAs indicated by miRPlant might suggest the hypothesis that some of the milRNAs generated by the mushroom are more closely related to plants than to animals. This observation might be in line with the fact that in nature, many fungi establish closer environmental relations with several trees and plants, forming mycorrhizas. The precise involvement of certain miRNAs in this symbiotic/parasitic nutrient exchange (besides secretion of certain signaling compounds) is not fully elucidated yet. In this sense, a different miRNA pattern is expressed in plants with or without mycorrhizas [40,41] and during infectious process [42]. In addition, fungal milRNAs have been indicated as key elements in this inter-kingdom talk [43], and technological approaches, also for siRNAs, to increase pest tolerance in plants have been suggested [23,24]. Therefore, it might be possible that fungal milRNAs would have needed to mimic plant miRNAs to enable a better fitting between two organisms belonging to two different kingdoms in a co-evolutionary process.

Beyond the former, and as previously mentioned, *A. bisporus* is one of the most produced mushrooms [34] and is part of the human diet. To this respect, since Zhang et al. reported that exogenous plant miRNAs were able to target the mammalian LDLRAP1 [44], an increasing number of papers have been published on this topic. For instance, the early research of Baier, Nguyen, Xie, Wood and Zempleni detected miR-29b and miR-200c, both bovine miRNAs [45], in plasma after cow milk intake, while Chin et al. proposed a cross-kingdom inhibition of breast cancer growth by plant miR159 [46]. Additionally, accumulating evidence indicates that sRNAs can be transferred within cells and tissues and even across species [47], and miRNAs from diet have been proposed to play a role on disease prevention [48,49]. Additionally, several authors report that dietary miRNAs can be absorbed with diet and reach their target mRNAs, both for animal [45,50] and plant [25,46] foodstuffs.

Although specific studies to clarify whether *A. bisporus* milRNAs could be absorbed with diet and reach their targets are needed, theoretical predictions can be useful for a non-blind search and to narrow and guide experimental research. In this respect, while abi_milRNAs are proposed to regulate basic metabolic pathways in *A. bisporus* (Table 4), they could be involved in the regulation of several pathological processes in humans. Thus, a third of the human pathways potentially regulated by abi-milRNAs are involved in diseases, i.e., 52 related to cancer, 28 to different infectious processes (including the infectious process by SARS-CoV-2: KEGG pathway hsa05171) and 7 to neurodegenerative diseases. Furthermore, we find the top 10 regulated pathways (Table 4) are of all of great relevance from a clinical perspective.

Moreover, it is wise to mention that recent publications pointed out with clinical studies the effect of specific mushrooms to prevent, for example, Alzheimer’s onset [51], or improve immune status in immunocompromised breast cancer patients [52]. *A. bisporus* extracts were also able to interfere with human prostate cancer cells [53]. In this respect, the above-mentioned diseases might potentially be regulated by abi_milRNAs, according to the theoretical prediction. For instance, abi-milRNAs may regulate approximately 16% of prostate cancer nodes, according to the KEGG Pathway database, leading again to the question if dietary miRNAs do or do not play a biological role such as phytochemicals do.

Overall, our data presented here provide evidence that the edible mushroom *Agaricus bisporus* contains miRNA-like small RNAs, with a collection of characteristics consistent with that of fungal miRNAs. In addition, *A. bisporus* milRNAs also share characteristics with miRNAs of plant origin more than with animal miRNAs, and, at least for some of the latest, experimental validation has been given. Finally, some pathways (both in mushrooms and in humans) are proposed to be (putatively) modulated by the presented *A. bisporus* milRNAs.

## 4. Materials and Methods

### 4.1. Biological Material

Fruiting bodies from *Agaricus bisporus* L. (Imbach) Fungisem H-15 were kindly offered by CTICH (Centro Tecnológico de Investigación del Champiñón de La Rioja, Autol, Spain) after cultivation under controlled conditions. Fruiting bodies from the first flush where harvested before their gills were exposed (developmental stage 2–3 according to Hammond and Nichols [54]. Afterwards, they were sliced, lyophilized and ground as described by Ramírez-Anguiano, Santoyo, Reglero and Soler-Rivas [55]. Mushroom caps and stipes were separated and prepared as indicated for the complete fruiting bodies. Mushroom powders were stored at −20 °C and in darkness until further use.

### 4.2. Small RNA Deep Sequencing

Small RNAs were extracted from powdered *A. bisporus* fruiting bodies using mirVanaTM miRNA Isolation Kit (Ambion^®^, Life Technologies, Huntingdon, UK) according to the manufacturer’s instructions, and quantified using a NanoDrop2000 (Thermofisher, Madrid, Spain). Then, further cDNA libraries and sequencing were performed. Briefly, low molecular RNAs were isolated by 15% TBE-urea denaturing polyacrylamide gel electrophoresis (PAGE) and ligated to specific adaptors (AGATCGGAAGAGCACACGTCT) at 3′ ends. After reverse transcription, appropriate amplification and purification, the cDNA was submitted to NGS single read 1 × 50 that was carried out using an Illumina MiSeq 2000 (Illumina^®^, Madrid, Spain). Raw sequencing data of sRNAs were deposited in the NCBI Sequence Read Archive (SRA) under accession no. PRJNA770841.

### 4.3. Data Analysis of Small RNA and miRNA Prediction

**Figure 7:**
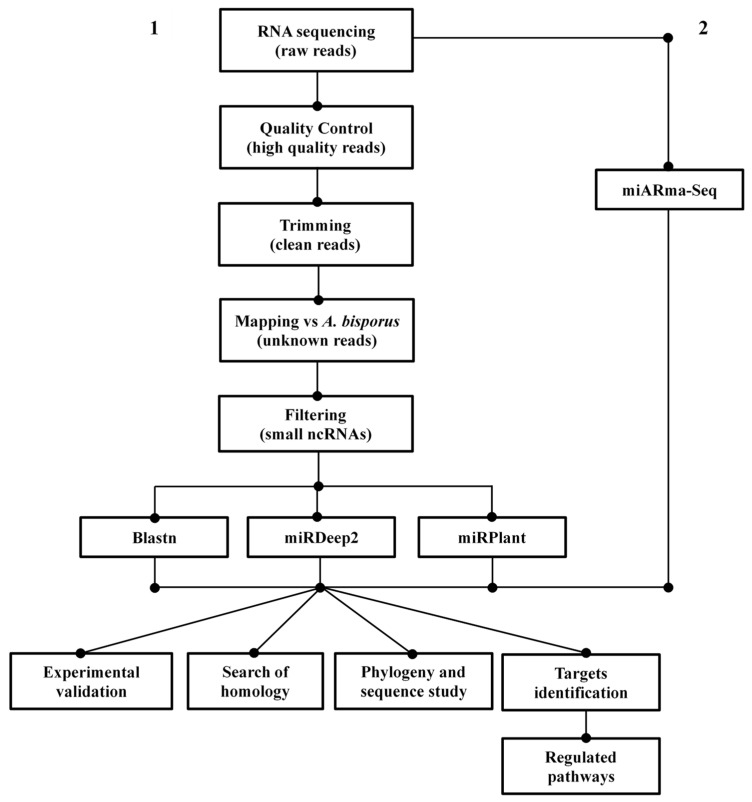
Flow-chart of the basic analysis process of milRNA sequencing data.

A preliminary bioinformatic analysis of sRNA data was carried out with the user-friendly miARma-Seq suite [56] (Figure 7(2)), followed by a step by step analysis for the identification of known and de novo miRNAs (Figure 7(1)).

First, quality control was conducted using FastQC [57], then raw reads were trimmed by stripping the adaptor sequence and removing reads with lengths below 18 nt using cutadapt [58]. Afterwards, clean reads were aligned against the representative genome of *A. bisporus* var. *bisporus* H97 (RefSeq assembly accession GCF_000300575.1) and downloaded from NCBI (https://www.ncbi.nlm.nih.gov/assembly/GCF_000300575.1, accessed on 9 February 2018), using bowtie 1.2.1 [59]. Read distribution within chromosomes was performed by mapping them toward *A. bisporus* var. H39 assembly (GCA_001682475.1), downloaded from NCBI (https://www.ncbi.nlm.nih.gov/assembly/GCA_001682475.1, accessed on 9 February 2018), where the 13 scaffolds corresponded to its 13 chromosomes. Python scripts were written to complete data analysis including total read counts, read frequency, etc.

The different ncRNAs (non-coding RNAs) types were determined and removed to filtered reads that might correspond to miRNAs. Thus, ncRNA files from Ensembl Fungi [60], NCBI (https://www.ncbi.nlm.nih.gov/gene, accessed on 30 March 2018), Silva [61], GtRNAdb [62], Rfam [63] including tRNA, snRNA, snoRNA and rRNA from 80S, 60S, 5S, 5.8S, 28S, 40S, 18S, LSU, SSU eukaryote families plus the Rfam file included in miRDeep2 [37] were downloaded and eliminated from the reads by using bowtie 1.2.1.

Annotation of filtered reads with their corresponding genomic regions (i.e., intergenic, exonic, intronic, etc.) was drawn by generating the corresponding *sam* files of perfect alignments obtained with bowtie 1. Then, they were converted into *bam* files with SAMtools [64] and from *bam* to *bed* files with BEDOPS v2.4.30 using the *bam2bed* command [65] to intersect them with the representative genome by using *intersect* from BEDTools [66]. The genome in *bed* file was obtained by rearranging the *gff* format columns with *awk* [67]. From the resulting file, the corresponding column was extracted with awk, and regions and frequency were counted with customized python scripts.

Although miARma and miRDeep2 identify known miRNAs by comparing the reads with known sequences included in miRBase using blast [68], specific nucleotide blast [69] with filtered reads against mature and pre-miRNAs (mature.fa and hairpin.fa) from miRBase21, release 21 [70], were also carried out to improve the control of the analysis parameters. Blasts were locally run with command *blastn–task blastn–short*, a word size of 8, more suitable for miRNA seed size [4] than the default value of 11 [71], and an e-value of 10^−4^ instead of 10, by default. Prediction of de novo miRNAs was carried out by miARma-seq and miRDeep2 (the latter is the central engine for miRNA processing in miARma-Seq and therefore they both run the same algorithm) using the *A. bisporus* var. *bisporus* H97 genome. An alternative prediction of de novo miRNAs based on criteria for plant biogenesis (instead of animal) was performed with miRPlant [38] selecting mature miRNA lengths between 18 to 26 nt. In this case *A. bisporus* var. H39 assembly was used, since a genome structured as chromosomes is required to run the program. Secondary structures suggested as pre-miRNAs were obtained online with RNAfold from Vienna RNA package [72]. A second check was performed by using milRNApredictor [73]. A tool specially designed to identify fungal milRNAs which core is the Randomforest algorithm.

Homologies between the predicted milRNAs in *A. bisporus* (known and de novo) and the sequences of 104 genomes from different Basidiomycetes species available in Ensembl Fungi (Table S4) were studied using Bowtie 1. A particular file containing most described fungal miRNAs was created to find out their potential relation with predicted de novo miRNAs. The file included data from *N. crassa* [11], *Fusarium oxysporum* [16], *Ganoderma lucidum* [19], *Trichoderma reesei* [14], *Penicillium marneffei* [15] and those obtained from *Pleurotus ostreatus* and *Lentinula edodes* (generated by the group but still unpublished). A multiple alignment was performed by T-Coffe [74] to obtain a phylogenic tree [75] with the default values of EMBL-EBI web. Moreover, a sequence logo for milRNAs from the white button mushroom was generated with Skylign [76].

### 4.4. miRNA Target Prediction

The potential targets of predicted miRNAs were pointed out using psRNATarget V2, release 2017 [35], and the representative *A. bisporus* genome. For human targets, psRNATarget V2 and miRAnda [31] were used and intersection between both were selected. For psRNATarget V2, default parameters were used, while for miRAnda a score cutoff (-sc) of 120, gap opening and gap extension (-go -ge) of -9 and -4, respectively, were used. In addition, a functional analysis of potential targets, on mushrooms and humans, was carried out by using the Mapper tool from Kyoto Encyclopedia of Genes and Genomes [33].

### 4.5. RT-qPCR Assay of miRNAs

sRNAs were extracted from mushroom caps and stipes using miRNeasy Serum/Plasma Kit (Qiagen, Madrid, Spain) according to the manufacturer’s protocol and quantified with a NanoDrop2000 (Thermofisher, Madrid, Spain). Retrotranscription (RT) was carried out using an miScript^®^ II RT (Qiagen, Madrid, Spain) kit, then the real-time PCR was used on a 7900HT Fast PCR (Applied Biosystems, Madrid, Spain) selecting 15 min at 95 °C, then 40 cycles of 15 s at 94 °C, 30 s at 50 °C and 45 s at 70 °C. Samples were mixed with the SYBR Green (Qiagen, Madrid, Spain) as fluorochrome. miScript Universal Primer (Qiagen, Madrid, Spain) for miRNAs and primers listed in Table S5 was used in this study. Their consensus mature sequence (CMS), consensus star sequence (CSS) and consensus precursor sequence (CPS) were evaluated using rRNA 5.8S as housekeeping RNA expression with the SYBR Green real-time PCR method. A no template control was used as negative control of real-time PCR. This negative control omits any DNA or RNA template from the reaction and serves as a general control for nucleic acid contamination or as control for primer dimer formation. An ANOVA and pair-wise Bonferroni tests were performed with R to discriminate differences between means corresponding to different samples.

## 5. Conclusions

The combination of NGS and bioinformatic tools allow the prediction of RNA regulatory elements, such as milRNAs. In addition, the characteristics of the predicted milRNAs are consistent with those described in other fungi and, for those that were experimentally validated, the metabolic precursors in the forms of pri-, pre- and mature ones were found, reinforcing the goodness of the prediction. Therefore, the molecular targets (in humans) are consistent with healthy properties previously reported, which along with their similarity with plant miRNAs leads them to be proposed as putative food components with healthy properties and to be experimentally validated in further research.

## Figures and Tables

**Table 1:** Composition of RNA library. Screening performed against an Ensembl Fungi file containing specific *A. bisporus* ncRNAs.

	Total Reads		Unique Reads	
	Number	%	Number	%
Raw reads	2,027,870	-	414,881	-
Trimmed reads (18–50 nt)	1,421,021	-	291,880	-
Mapped reads	1,015,249	100.00	117,838	100.00
rRNA	94,076	9.26	1786	1.52
tRNA	267,465	26.45	3472	2.95
snRNA	26,952	2.65	1823	1.55
snoRNA	179	0.02	80	0.07
Unknown reads	626,577	61.72	110,677	93.92

**Table 2:** *Agaricus bisporus* milRNA (microRNA-like) candidates predicted by miRDeep2 (and miARma-Seq) and miRPlant. Reads correspond to number of reads. L: length. Pre-miRNA position corresponds to H97 assembly for miRDeep2 group and to H39 for miRPlant one.

**miRDeep2**				
**Name**	**Sequence 5′→3′**	**Reads**	**L**	**Pre-miRNA Position**
abi_milRNA_1a_1	GUGGGCUGGGCUGCUGCAGCG	38,803	21	Scaffold_10: 1624834...1624880: +
abi_milRNA_1a_2	GUGGGCUGGGCUGCUGCAGCG	38,803	21	Scaffold_10: 1633264...1633310: +
abi_milRNA_1a_3	GUGGGCUGGGCUGCUGCAGCG	38,803	21	Scaffold_10: 1586743...1586789: +
abi_milRNA_1a_4	GUGGGCUGGGCUGCUGCAGCG	38,803	21	Scaffold_10: 1615990...1616035: +
abi_milRNA_2a	UCUAAUCAUGGACGUGCU	1835	18	Scaffold_19: 127065...127112: −
abi_milRNA_3a	UCAGCUCGCAAUGUAGAUAUU	1186	21	Scaffold_9: 984246...984325: −
abi_milRNA_4a	AGGCUGCGGAACGUUGGCACGGGU	34	24	Scaffold_8: 1573143...1573195: −
abi_milRNA_5a	UGACUUAGGACGACCCGCCA	10	20	Scaffold_5: 515832...515871: +
abi_milRNA_6a	GGCGAGAUGGCCGAGUGGUCU	48	21	Scaffold_7: 612160...612226: −
**miRPlant**				
**Name**	**Sequence 5′→3′**	**Reads**	**L**	**Pre-miRNA Position**
abi_milRNA_7a	GGUUGCGUCGGGGAACCAGGACU	62,926	23	Ch9(+): 1609844...1610066
abi_milRNA_8a_1	GGCCGACUAGCUCAGUUGGU	9443	20	Ch8(−): 1458375...1458557
abi_milRNA_8a_2	GGCCGACUAGCUCAGUUGGU	9429	20	Ch12(+): 1449317...1449402
abi_milRNA_9a	UCUCUGUUAGUAUAUCGGU	7428	19	Ch13(−): 409450...409650
abi_milRNA_9b	UCUCUGUUAGUAUAUCGGUUAGU	1580	24	Ch1(+): 595123...595304
abi_milRNA_10a	UUUUCCUGUGAAGCAUGUUCU	3570	21	Ch7(−): 2126076...2126293
abi_milRNA_11a	UCGACUGUUGUAUCCUUUGCA	1784	21	Ch7(−): 577587...577706
abi_milRNA_12a_1	CCGACCUUAGCUCAGUUGGAAGA	1301	23	Ch5(+): 1529471...1529665
abi_milRNA_12a_2	CCGACCUUAGCUCAGUUGGAAGA	314	23	Ch9(+): 1638595...1638793
abi_milRNA_12a_3	CCGACCUUAGCUCAGUUGGAAGA	1782	23	Ch5(+): 1529623...1529826
abi_milRNA_12a_4	CCGACCUUAGCUCAGUUGGAAGA	1781	23	Ch12(−): 118842...118950
abi_milRNA_12a_5	CCGACCUUAGCUCAGUUGGAAGA	1781	23	Ch7(+): 1669558...1669699
abi_milRNA_13a_1	CUAGUGGUUAUGAUUUCUGUCU	1073	22	Ch10(+): 317766...317943
abi_milRNA_13a_2	CUAGUGGUUAUGAUUUCUGUCU	832	22	Ch10(+): 206312...206533
abi_milRNA_13a_3	CUAGUGGUUAUGAUUUCUGUCU	832	22	Ch10(−): 208566..208671
abi_milRNA_13a_4	CUAGUGGUUAUGAUUUCUGUCU	1040	22	Ch10(+): 206312...206533
abi_milRNA_14a	UUAGUGGUUAGAUCAUCUCGUU	1001	22	Ch12(−): 153917...154009
abi_milRNA_15a	GUGUAGUGGUUAUCACUCGGGAUU	593	24	Ch7(−): 874207...874386
abi_milRNA_16a	UAAGCCCUUGUUCUAUAGAUUUGU	627	24	Ch9(+): 1685049...1685150
abi_milRNA_17a	GGGUAGUGGUAACCUGGGUCGUUG	431	24	Ch12(−): 301528...301656
abi_milRNA_18a_1	UCGGAACCCGCUAAGGAGUGUG	335	22	Ch9(+): 1655122...1655321
abi_milRNA_18a_2	UCGGAACCCGCUAAGGAGUGUG	314	22	Ch9(+): 1604213...1604411
abi_milRNA_18a_3	UCGGAACCCGCUAAGGAGUGUG	314	22	Ch9(+): 1612977..1613175
abi_milRNA_18a_4	UCGGAACCCGCUAAGGAGUGUG	314	22	Ch9(+): 1621357...1621555
abi_milRNA_18a_5	UCGGAACCCGCUAAGGAGUGUG	314	22	Ch9(+): 1630532...1630730
abi_milRNA_18a_6	UCGGAACCCGCUAAGGAGUGUG	314	22	Ch9(+): 1638595...1638793
abi_milRNA_18a_7	UCGGAACCCGCUAAGGAGUGUG	314	22	Ch9(+): 1665017...1665215
abi_milRNA_18a_8	UCGGAACCCGCUAAGGAGUGUG	314	22	Ch9(+): 1672337...1672535
abi_milRNA_18a_9	UCGGAACCCGCUAAGGAGUGUG	314	22	Ch9(+): 1682088...1682286
abi_milRNA_19a	ACACUGACAGAGCCAGCGAGUUUU	191	24	Ch9(+): 1628437..1628530
abi_milRNA_20a_1	UAUAGUUUAUUUGAUGAUACCU	186	22	Ch9(+): 1668929...1669062
abi_milRNA_20a_2	UAUAGUUUAUUUGAUGAUACCU	186	22	Ch9(+): 1617899...1168030
abi_milRNA_20a_3	UAUAGUUUAUUUGAUGAUACCU	186	22	Ch9(+): 1627076...1627207
abi_milRNA_20a_4	UAUAGUUUAUUUGAUGAUACCU	186	22	Ch9(+): 1650974...1651105
abi_milRNA_20a_5	UAUAGUUUAUUUGAUGAUACCU	186	22	Ch9(+): 1659680...1659791
abi_milRNA_20a_6	UAUAGUUUAUUUGAUGAUACCU	186	22	Ch9(+): 1661558...1661689
abi_milRNA_20a_7	UAUAGUUUAUUUGAUGAUACCU	186	22	Ch9(+): 1678625...1678756
abi_milRNA_21a	GUGUAGCGGUAACAUUGGGUCUU	80	23	Ch5(+): 727119...727291
abi_milRNA_22a	UUGCCCGACCAUGUAGCCUU	74	20	Ch2(−): 427069...427198
abi_milRNA_23a	GUCACUUUGCCGGAGUGGUUAAC	70	23	Ch3(+): 2279394...2279576
abi_milRNA_24a	CACCACGGACGGUCUGUAGCUCCU	68	24	Ch13(+): 1171206...1171324
abi_milRNA_25a	GCUGGGACUGCUGUGGUU	30	18	Ch1(−): 3410229...3410399
abi_milRNA_26a	UGUGAUCUGGAUUGGAACAUUC	27	22	Ch1(+): 2058804...2058899
abi_milRNA_27a	GGACCCCUAGCUCAGUGG	20	18	Ch2(−): 9192...9296
abi_milRNA_27b	GGACCCCUAGCUCAGUGGUAGA	13	22	Ch2(−): 9255...9354
abi_milRNA_28a	UGUGGUCAUCUUAGAGCUCACU	20	22	Ch2(+): 2696324...2696545
abi_milRNA_29a_1	UUACGUGGCUCAAGGGUUAAG	15	21	Ch2(+): 347744...347918
abi_milRNA_29a_2	UUACGUGGCUCAAGGGUUAAG	11	21	Ch2(+): 347878...347959
abi_milRNA_30a	AGUGGACUUGGCAUGCGAGAGGUU	15	24	Ch12(+): 530586...530694
abi_milRNA_31a	UGCCUUCAUUGGAUCGUGCU	15	20	Ch3(−): 1354888...1354997
abi_milRNA_32a	GCUGUACUCAUUUCUGUAU	12	19	Ch2(−): 2797465...2797543
abi_milRNA_33a_1	CCAAACGAUCUAAUCCAGAACU	11	22	Ch4(−): 63633...63729
abi_milRNA_33a_2	CCAAACGAUCUAAUCCAGAACU	10	22	Ch4(−): 63688...63909
abi_milRNA_34a	UAUAGUACUAAGAGCUUGAGAGU	10	23	Ch9(+): 1090217...1090326
abi_milRNA_35a	UAUCGACGUACACUUAUUGGU	10	21	Ch10(+): 127320...127540
abi_milRNA_36a	CGAUCGGCGAUAUCGAGACUA	9	21	Ch7(−): 69025...69107
abi_milRNA_37a	GCUAGCGUGCUUACUACUGUA	7	21	Ch4(+): 1307833...1307940

**Table 3:** Fungal miRNAs with experimental evidence and their potential orthologues.

miRNA Source	miRNAs with Experimental Evidence	miRNAs Sharing an Ancestor	Reference
*Agaricus bisporus*	abi_milRNA_2a	abi_milRNA_31a	Present work
*Agaricus bisporus*	abi_milRNA_1a	abi_milRNA_25a, pos_milRNA_6a	Present work
*Fusarium oxysporum*	fox_milRNA_2a	abi_milRNA_6a abi_milRNA_17a	[16]
*Fusarium oxysporum*	fox_milRNA_5	pos_milRNA_5a	[16]
*Penicillium marneffei*	PM_milR_M1	abi_milRNA_22a	[15]
*Penicillium marneffei*	PM_milR_M2	abi_milRNA_26a	[15]

**Table 4:** Top 10 regulated pathways, by proposed abi_milRNAs, in *A. bisporus* and *H. sapiens* according to KEGG.

*Agaricus bisporus*			*Homo sapiens*		
Regulated Pathway	Genes	KEGG Code	Regulated Pathway	Genes	KEGG Code
Metabolic pathways	304	abv01100	Metabolic pathways	91	hsa01100
Biosynthesis of secondary metabolites	124	abv01110	Pathways in cancer	51	hsa05200
Biosynthesis of cofactors	50	abv01240	Herpes simplex virus 1 infection	39	hsa05168
Cell cycle	40	abv04111	Pathways of neurodegeneration—multiple diseases	38	hsa05022
Carbon metabolism	35	abv01200	Human papillomavirus infection	31	hsa05165
Autophagy	33	abv04138	MAPK signaling pathway	30	hsa04010
MAPK signaling pathway	32	abv04011	PI3K–Akt signaling pathway	25	hsa04151
Protein processing in endoplasmic reticulum	32	abv04141	Endocytosis	25	hsa04144
Nucleocytoplasmic transport	31	abv03013	Salmonella infection	25	hsa05132
Spliceosome	31	abv03040	Shigellosis	24	hsa05132

## Data Availability

Data supporting the reported results can be found at the following link: https://www.ncbi.nlm.nih.gov/bioproject/PRJNA770841, accessed on 13 October 2021.

## Supplementary Materials

The following supporting information can be downloaded at: https://www.mdpi.com/article/10.3390/ijms23094923/s1.
