# Supplementary material for: RNA-Seq, Bioinformatic Identification of Potential MicroRNA-like Small RNAs in the Edible Mushroom Agaricus bisporus and Experimental Approach for Their Validation
Source: Int J Mol Sci. 2022 Apr 28;23(9):4923. doi: 10.3390/ijms23094923 (PMC9100230; doi:10.3390/ijms23094923)
Supplement: Supplementary file 1 [file ijms-23-04923-s001.zip › ijms-1667877-supplementary.pdf]

International Journal of
Molecular Sciences

MDPI

Suplementary Material

Table S1. Screening against different classes of ncRNAs from different databases. In the second column, the number between brackets indicates file features: **1.** Rfam file downloaded from miRDeep2. **2.** Rfam file corresponding to different RNA classes (i.e. snRNA, tRNA, etc) of *Agaricus bisporus*. **3.** Ensembl Fungi file corresponding to different RNA classes of *Agaricus bisporus*. **4.** NCBI-Nucleotides file corresponding to different RNA classes of *Fungi*. **5.** Raw files from other databases without refining their contents. For instance, Silva file contains all available rRNA without attending taxonomic group.

RNA class	Database	Total reads	Unique reads
Mapped reads		1015249 (100.00 %)	117838 (100.00 %)
tRNA	Rfam (1)	23968 (2.36%)	840 (0.71 %)
	Rfam (2)	0 (0.00%)	0 (0.00%)
	Ensembl_Fungi (3)	267465 (26.45%)	3472 (2.94 %
	NCBI (4)	1 (0.00%)	1 (0.00 %)
	GtRNA (5)	1119 (0.11%)	120 (0.10 %)
snRNA	Rfam (2)	0 (0.00%)	0 (0.00%)
	Ensembl_Fungi (3)	26952 (2.65 %)	1823 (1.54%)
	NCBI (4)	1634 (0.16 %)	87 (0.07 %)
	SILVA (5)	11 (0.00 %)	8 (0.01 %)
snoRNA	Rfam (2)	0 (0.00%)	0 (0.00%)
	Ensembl_Fungi (3)	179 (0.02 %)	80 (0.07%)
	NCBI (4)	00 (0.00%)	0 (0.00 %)
	SILVA (5)	1 (0.00%)	1 (0.00 %)
rRNA	Rfam (1)	119153 (11.74 %)	4217 (3.59 %)
	Rfam (2)	13 (0.00 %)	11 (0.01 %)
	Ensembl_Fungi (3)	94076 (9.26 %)	1786 (1.52%)
	NCBI (4)	259581 (25.56 %)	18174 (15.42%)
	SILVA (5)	55 (0.01%)	8 (0.01 %)
ncRNA	miRDeep (1)	136120 (1.29 %)	5057 (4.29%)
	Rfam (2)	13 (0.00 %)	11 (0.01)
	Ensembl_Fungi (3)	388675 (38.42 %)	7164 (6.08 %)
	NCBI (4)	261201 (25.72 %)	18253 (15.49 %)
lncRNA	NONCODE (5)	109 (0.01 %)	84 (0.07 %)

Table S2. *Agaricus bisporus* pre-milRNAss (Micro-Like-RNAs) candidates predicted by miRDeep2 (and miARma-Seq) and miRPlant. Mature milRNA sequence is shown in capital letters.

	**miRDeep2**
**Name**	**Sequence 5’ →3’**
pre-abi_milRNAs_1a	GUGGGCUGGGCUGCUGCAGCGgaaaugcuguggugggcuuguccacg
pre-abi_milRNAs_2a	uagcucagugguagagcgugagauuccagUCUAAUCAUGGACGUGCU
pre-abi_milRNAs_3a	ucacuucguuguggcuugugcuccgaagagccgcugguucgauuccagcuacggacacUCAGCUCGCAAUGUAGA
pre-abi_milRNAs_4a	uacugucgccauuggaaaacagcuuucuAGGCUGCGGAACGUUGGCACGGGU
pre-abi_milRNAs_5a	cggauuguccaagucguuuUGACUUAGGACGACCCGCCA
pre-abi_milRNAs_6a	auggcucccauguccaagugcauucgacgaggcauugguugugcaGGCGAGAUGGCCGAGUGGUCU
	**miRPlant**
**Name**	**Sequence 5’ →3’**
pre-abi_milRNAs_7a	ggccuucacuGGUUGCGUCGGGGAACCAGGACUuuuaccuugagaaaauuagaguguucaaagcaggccuaugccugaauacauuagcauggaauaauaaaauaggacgugcgguucuauuuuguugguuucuagagucgccguaaugauuaauagggauaguugggggcauugguauugagucgcuagaggugaaauucuuggauugacuccuuuuuagug
pre-abi_milRNAs_8a	cugcuugaaccacugauccugggaacuuaccauaagaccacaauuugugauaugcaGGCCGACUAGCUCAGUUGGUuagagcgucg
pre-abi_milRNAs_9a	aguacuuaaccgaaauacagguggcagaaaaaguaaaacauugaucgagcaugcuaaaaugacaguaucuuugaaugaaggguuuaug uggcgcuuuuccaacagccggagacaagcucaagacuagcuagcuauuuggaaugaUCUCUGUUAGUAUAUCGGUuaguacacgc
pre-abi_milRNAs_9b	aauugaguacuuaaccgaaauacagguggcagaaaaaguaaaacauugaucgagcaugcuaaaaugacaguaucuuugaaugaaggguuuauguggcgcuuuuccaacagccggagacaagcucaagacuagcuagcuauuuggaaugaUCUCUGUUAGUAUAUCGGUUAGUacacgcgcuu
pre-abi_milRNAs_10a	ugcggcgucuggcauagcuguagcaaggauaagcaauaauaaacauaauuuggcccgccuugcucucguaacuguuguauuggacuauuugguauuuacuccaaggcgcuauagccaagugguuacggcgacagauuagaaaucuguuccucuuugagggcgcagguucgaauccugcuggcgucgcUUUUCCUGUGAAGCAUGUUCUuuuucuacca
pre-abi_milRNAs_11a	ucgauggauaUCGACUGUUGUAUCCUUUGCAcgacauuuugugucuauggcgcgcuucgaguccgaagggaccaugccgggcgucuugcgauggauggcuacagucgauauccaaguugu
pre-abi_milRNAs_12a	ugauuaacaaCCGACCUUAGCUCAGUUGGAAGAgcgucagacuguaauuggauaacuugauuuucaucugaaugucgccuguucgaaccaggcaggucggacuaaucugauc
pre-abi_milRNAs_13a	uaaaaaagagaauaagaaaugaauuggaugagcguugggcaacggaugaucguugaugcgaauggucgaaugguCUAGUGGUUAUGAUUUCUGUCUcacacacaga
pre-abi_milRNAs_14a	agguaauuauguuugugaucuggcauugcuaauuugcuuugucucuauacguccuugucgaUUAGUGGUUAGAUCAUCUCGUUguggucacuu
pre-abi_milRNAs_15a	gaggugcauauuccggauggugauaaggaugcgugaaguguugggaagacaucccaagggaaaaagaaaguuggcgugggcguuuggaaaguaugugcagcguccauguuauaaacacauugaaaauaaauggcauggcguuauaGUGUAGUGGUUAUCACUCGGGAUUuugaucguag
pre-abi_milRNAs_16a	gcagacgacugaaugggaacgggguacuguaagcgguagaguagccuguugcuacgauccgcugagguUAAGCCCUUGUUCUAUAGAUUUGUucaacauuuc
pre-abi_milRNAs_17a	caucgagaagaggaccgaucaagugagccaaguguguaucugcggugucgcugcugauggcccuaaaacgagcaguagcguugcgagcauuaaugGGGUAGUGGUAACCUGGGUCGUUGccaucugcgc
pre-abi_milRNAs_18a	uugauugaaccgcucggcgauugagaguuucuaguggggccauuuuugguaagcagaacuggcgaugcgggaugaaccgaacgcgagguuaaggugccggaauucacgcucaucagacaccacaaaagguguuaguucaucuagacagcaggacgguggccauggaagUCGGAACCCGCUAAGGAGUGUGuaacaacuca
pre-abi_milRNAs_19a	cacgcgcgcuACACUGACAGAGCCAGCGAGUUUUucaccuuggccggaaggucugggguaaucuugugaaacucugucgugcuggggauagagca

pre-abi_milRNAs_20a	uuaaaucaguUAUAGUUUAUUUGAUGAUACCUugcuacauggauaacugugguaauucuagagcuaauacaugcaaucaagccccgacuuccggaaggggguguauuuauuagauaaaaaaccaacgcgguuc
pre-abi_milRNAs_21a	augaccuuggauuccaaucggugaccguuuaucgucgauaguagacaagguuuauuguuauagaugaaagcucuuucucaacgugaaacacaaaaacauauauacgguuuaugagcgcauuacuuuuguaggcauugaugGUGUAGCGGUAACAUUGGGUCUUcccauggccu
pre-abi_milRNAs_22a	agguauuuguugaugguagaagcauugaugguuuaaugguaaccaacgucgucgucgaucgcguucgacgugcuggagguucgacuccuccuugaugcagUUGCCCGACCAUGUAGCCUUuuuaaaaaac
pre-abi_milRNAs_23a	cguucaguaaGUCACUUUGCCGGAGUGGUUAACgggaucgccugcuacuuaauguggcugauuagcgguuggucuuugaccgcgaguguucgaaucgcucaggugacggaauuuuuuucuuguuauuucuugauuuucugcacccgcguuccguaggaucagguaacagacggauuguuugca
pre-abi_milRNAs_24a	uugugauuccCACCACGGACGGUCUGUAGCUCCugccaagcauaguuuuccuuggcuuuugccaauaagaaugaaacuuaacaugagcuagcaaaccugccggggguaggaaaauaucaa
pre-abi_milRNAs_25a	ugacgccaguccgccagucauucccgcaaagaaaccuuccuuccgggcuuccuguaggcgaauaugguugacguugagcagggcaaggccgaggagaaacuugccuugagaacgcgagccucugcgucaggaggaagguccgaGCUGGGACUGCUGUGGUUguuguccaug
pre-abi_milRNAs_26a	gggacgcagauugaucuucuccuucugcaucacgaggaacugugcaaucguaggcauagggcuaUGUGAUCUGGAUUGGAACAUUCauacauuaua
pre-abi_milRNAs_27a	acauauacgaGGACCCCUAGCUCAGUGGuagagcgugagauuccaguuuaugcguguuuccucaaggucauccguucaauccggguggggguucacuuuuuccaaa
pre-abi_milRNAs_27b	uuucccacaucaucacuuucguuaaagucccguucucaguuauacaauggagagauaaacauauacgaGGACCCCUAGCUCAGUGGUAGAgcgugagauu
pre-abi_milRNAs_28a	acugauuugaUGUGGUCAUCUUAGAGCUCACUcucgaugcccuagaccauucuccucgggggucagagccgguagaccguacaccguagaucuuuauaaaucucuugucacuuucuuucucugagacaucuguuuaaaggccuugcgcgauccuuuuccugucacccucaucuaccccuacuuucuuggauauccugccugaagaugacgaccguccucggu
pre-abi_milRNAs_29a	acuacuuacuuggcuuuaaguccaaugacgugaucaaagguuuuguauuuuguucagcccgcggggguaccgucacgugaucuaaaauccgcugacaucagucauggggguauauggugcacuaagagaaauaaguauuaagcuagUUACGUGGCUCAAGGGUUAAGuaauuuauug
pre-abi_milRNAs_30a	ugucagcaugAGUGGACUUGGCAUGCGAGAGGUUuugguuuggugguagaugccauguuuauauccaaucuguaacuaccucccaugcuuauccgaguugcuucaucua
pre-abi_milRNAs_31a	ccacguaguuUGCCUUCAUUGGAUCGUGCUuaugucuuuagguucaauuguaauuuuuuugcucccuaucauugcgcacgaaucuaagaaugaacgcuauuccuaauuua
pre-abi_milRNAs_32a	cggugcuauaGCUGUACUCAUUUCUGUAUaacaucaucgauugacuagugcuacgggaguaaguggcagagcacggcaa
pre-abi_milRNAs_33a	caacggaacuCCAAACGAUCUAAUCCAGAACUugaaccccauagcuaucaucauaaugguaccuuucuuugauuaugucguuuacccgcuacuccgc
pre-abi_milRNAs_34a	uagaaauacucagaguucuaaagcguaacaaaggagaccuccucaaugaaucgagaagaguaacucaaaugcacuguUAUAGUACUAAGAGCUUGAGAGUgguuuauaga
pre-abi_milRNAs_35a	aaugaaaaaaUAUCGACGUACACUUAUUGGUagcggcgagguucgaacucgcgcggacuuucgcccauugggaaccgaaacacggaacuuaaauccaacgccuuaaaccacucggccacgcuaccguccaaaugucagagucuuugugguucuauagggagauuuguuuguagcucguagucgaucuaaacgaugcccaccgagguaugcgguaaauaugau
pre-abi_milRNAs_36a	acacaucccaCGAUCGGCGAUAUCGAGACUAucuugaauauaugugucaagagccucgaaucugcugaucaacucacucguca
pre-abi_milRNAs_37a	cuaagcaaguGCUAGCGUGCUUACUACUGUAaccguuuuguuuugacugcaccccauaguuucaaguugaugaguacagcguaugcauacccuagcuuuuuuuguaaa

Table S3. Homology between 37 *de novo* predicted *Agaricus bisporus* milRNAss and 104 *Basidiomycetes* species. Results show number and percentage (between brackets) for 0, 1 and 2 mismatches (from left to right, 2nd, 3rd and 4th columns). In column to the right (milRNAss) are listed those milRNAss with 0 or 1 mismatch. Species are listed in alphabetical order.

	mismatchs			
**Species**	**0**	1	**2**	**milRNAss**
Amanita muscaria	8 (21.05%)	10 (26.32%)	15 (39.47%)	abi_milRNAs_13a abi_milRNAs_8a abi_milRNAs_23a abi_milRNAs_18a abi_milRNAs_9b abi_milRNAs_17a abi_milRNAs_21a abi_milRNAs_15a abi_milRNAs_6a
Anthracocystis flocculosa	0 (0.00%)	3 (7.89%)	7 (18.42%)	abi_milRNAs_25a abi_milRNAs_19a abi_milRNAs_6a
Botryobasidium botryosum	1 (2.63%)	6 (15.79%)	12 (31.58%)	abi_milRNAs_8a abi_milRNAs_18a abi_milRNAs_14a abi_milRNAs_19a abi_milRNAs_17a abi_milRNAs_6a
Calocera cornea	0 (0.00%)	1 (2.63%)	6 (15.79%)	abi_milRNAs_23a
Calocera viscosa	1 (2.63%)	3 (7.89%)	6 (15.79%)	abi_milRNAs_25a abi_milRNAs_23a abi_milRNAs_18a
Ceraceosorus bombacis	0 (0.00%)	1 (2.63%)	5 (13.16%)	abi_milRNAs_16a
Coniophora puteana	8 (21.05%)	11 (28.95%)	18 (47.37%)	abi_milRNAs_13a abi_milRNAs_25a abi_milRNAs_8a abi_milRNAs_23a abi_milRNAs_18a abi_milRNAs_19a abi_milRNAs_17a

				abi_milRNAs_7a abi_milRNAs_21a abi_milRNAs_15a abi_milRNAs_16a
Coprinopsis cinerea	1 (2.63%)	2 (5.26%)	3 (7.89%)	abi_milRNAs_23a abi_milRNAs_16a
Cryptococcus amylolentus	0 (0.00%)	1 (2.63%)	4 (10.53%)	abi_milRNAs_18a
Cryptococcus depauperatus	0 (0.00%)	0 (0.00%)	0 (0.00%)	-----
Cryptococcus gattii	0 (0.00%)	0 (0.00%)	1 (2.63%)	-----
Cryptococcus neoformans	1 (2.63%)	2 (5.26%)	8 (21.05%)	abi_milRNAs_18a abi_milRNAs_16a
Cutaneotrichosporon oleaginosus	1 (2.63%)	1 (2.63%)	3 (7.89%)	abi_milRNAs_6a
Cylindrobasidium torrendii	3 (7.89%)	8 (21.05%)	11 (28.95%)	abi_milRNAs_13a abi_milRNAs_8a abi_milRNAs_23a abi_milRNAs_9b abi_milRNAs_17a abi_milRNAs_15a abi_milRNAs_6a
Dacryopinax primogenitus	1 (2.63%)	4 (10.53%)	8 (21.05%)	abi_milRNAs_23a abi_milRNAs_20a abi_milRNAs_18a abi_milRNAs_31a
Daedalea quercina	7 (18.42%)	9 (23.68%)	15 (39.47%)	abi_milRNAs_8a abi_milRNAs_23a abi_milRNAs_20a abi_milRNAs_9b abi_milRNAs_17a abi_milRNAs_21a abi_milRNAs_15a abi_milRNAs_16a abi_milRNAs_9b

Dichomitus squalens	9 (23.68%)	11 (28.95%)	14 (36.84%)	abi_milRNAs_8a<br>abi_milRNAs_23a<br>abi_milRNAs_9b<br>abi_milRNAs_14a<br>abi_milRNAs_19a<br>abi_milRNAs_17a<br>abi_milRNAs_21a<br>abi_milRNAs_15a<br>abi_milRNAs_16a<br>abi_milRNAs_9b<br>abi_milRNAs_6a
Exidia glandulosa	0 (0.00%)	0 (0.00%)	3 (7.89%)	-----
Fibroporia radiculosa	4 (10.53%)	9 (23.68%)	13 (34.21%)	abi_milRNAs_8a<br>abi_milRNAs_23a<br>abi_milRNAs_18a<br>abi_milRNAs_9b<br>abi_milRNAs_14a<br>abi_milRNAs_17a<br>abi_milRNAs_21a<br>abi_milRNAs_15a<br>abi_milRNAs_9b
Fibulorhizoctonia spp	3 (7.89%)	9 (23.68%)	12 (31.58%)	abi_milRNAs_25a<br>abi_milRNAs_8a<br>abi_milRNAs_23a<br>abi_milRNAs_18a<br>abi_milRNAs_19a<br>abi_milRNAs_17a<br>abi_milRNAs_7a<br>abi_milRNAs_16a<br>abi_milRNAs_6a
Fistulina hepatica	2 (5.26%)	7 (18.42%)	11 (28.95%)	abi_milRNAs_13a<br>abi_milRNAs_8a<br>abi_milRNAs_23a<br>abi_milRNAs_14a

				abi_milRNAs_17a<br>abi_milRNAs_15a<br>abi_milRNAs_6a
Fomitiporia mediterranea	2 (5.26%)	11 (28.95%)	16 (42.11%)	abi_milRNAs_13a<br>abi_milRNAs_25a<br>abi_milRNAs_23a<br>abi_milRNAs_18a<br>abi_milRNAs_19a<br>abi_milRNAs_17a<br>abi_milRNAs_15a<br>abi_milRNAs_16a<br>abi_milRNAs_9b<br>abi_milRNAs_6a
Fomitopsis pinicola	6 (15.79%)	7 (18.42%)	12 (31.58%)	abi_milRNAs_8a<br>abi_milRNAs_23a<br>abi_milRNAs_9b<br>abi_milRNAs_17a<br>abi_milRNAs_21a<br>abi_milRNAs_15a<br>abi_milRNAs_9b
Galerina marginata	10 (26.32%)	14 (36.84%)	20 (52.63%)	abi_milRNAs_13a<br>abi_milRNAs_27a<br>abi_milRNAs_8a<br>abi_milRNAs_23a<br>abi_milRNAs_20a<br>abi_milRNAs_9b<br>abi_milRNAs_19a<br>abi_milRNAs_27b<br>abi_milRNAs_17a<br>abi_milRNAs_21a<br>abi_milRNAs_15a<br>abi_milRNAs_16a<br>abi_milRNAs_9b

				abi_milRNAs_31a
Ganoderma lucidum	8 (6.20%)	11 (8.53%)	15 (11.63%)	abi_milRNAs_8a<br>abi_milRNAs_23a<br>abi_milRNAs_14a<br>abi_milRNAs_17a<br>abi_milRNAs_21a<br>abi_milRNAs_15a<br>abi_milRNAs_16a<br>abi_milRNAs_9b<br>abi_milRNAs_6a
Gelatoporia subvermispora	9 (23.68%)	14 (36.84%)	18 (47.37%)	abi_milRNAs_25a<br>abi_milRNAs_8a<br>abi_milRNAs_23a<br>abi_milRNAs_20a<br>abi_milRNAs_18a<br>abi_milRNAs_9b<br>abi_milRNAs_14a<br>abi_milRNAs_19a<br>abi_milRNAs_17a<br>abi_milRNAs_21a<br>abi_milRNAs_15a<br>abi_milRNAs_16a<br>abi_milRNAs_9b<br>abi_milRNAs_6a
Gloeophyllum trabeum	4 (10.53%)	7 (18.42%)	15 (39.47%)	abi_milRNAs_13a<br>abi_milRNAs_8a<br>abi_milRNAs_23a<br>abi_milRNAs_20a<br>abi_milRNAs_17a<br>abi_milRNAs_21a<br>abi_milRNAs_6a
Grifola frondosa	5 (13.16%)	10 (26.32%)	15 (39.47%)	abi_milRNAs_8a<br>abi_milRNAs_23a<br>abi_milRNAs_20a

				abi_milRNAs_18a<br>abi_milRNAs_9b<br>abi_milRNAs_14a<br>abi_milRNAs_17a<br>abi_milRNAs_21a<br>abi_milRNAs_15a<br>abi_milRNAs_16a
Gymnopus luxurians	3 (7.89%)	8 (21.05%)	14 (36.84%)	abi_milRNAs_8a<br>abi_milRNAs_23a<br>abi_milRNAs_20a<br>abi_milRNAs_18a<br>abi_milRNAs_19a<br>abi_milRNAs_17a<br>abi_milRNAs_16a<br>abi_milRNAs_6a
Hebeloma cylindrosporum	10 (26.32%)	14 (36.84%)	20 (52.63%)	abi_milRNAs_13a<br>abi_milRNAs_27a<br>abi_milRNAs_8a<br>abi_milRNAs_23a<br>abi_milRNAs_20a<br>abi_milRNAs_18a<br>abi_milRNAs_27b<br>abi_milRNAs_17a<br>abi_milRNAs_21a<br>abi_milRNAs_15a<br>abi_milRNAs_16a<br>abi_milRNAs_9b<br>abi_milRNAs_31a
Heterobasidion irregulare	5 (13.16%)	10 (26.32%)	17 (44.74%)	abi_milRNAs_13a<br>abi_milRNAs_8a<br>abi_milRNAs_23a<br>abi_milRNAs_20a

				abi_milRNAs_18a<br>abi_milRNAs_17a<br>abi_milRNAs_15a<br>abi_milRNAs_9b<br>abi_milRNAs_6a
Hydnomerulius pinastri	5 (13.16%)	6 (15.79%)	12 (31.58%)	abi_milRNAs_13a<br>abi_milRNAs_8a<br>abi_milRNAs_23a<br>abi_milRNAs_17a<br>abi_milRNAs_15a<br>abi_milRNAs_21a
Hypholoma sublateritium	9 (23.68%)	11 (28.95%)	16 (42.11%)	abi_milRNAs_13a<br>abi_milRNAs_27a<br>abi_milRNAs_8a<br>abi_milRNAs_23a<br>abi_milRNAs_9b<br>abi_milRNAs_27b<br>abi_milRNAs_17a<br>abi_milRNAs_21a<br>abi_milRNAs_15a<br>abi_milRNAs_9b<br>abi_milRNAs_31a
Hypsizygus marmoreus	4 (10.53%)	7 (18.42%)	14 (36.84%)	abi_milRNAs_13a<br>abi_milRNAs_8a<br>abi_milRNAs_23a<br>abi_milRNAs_14a<br>abi_milRNAs_17a<br>abi_milRNAs_21a<br>abi_milRNAs_15a
Jaapia argillacea	5 (13.16%)	7 (18.42%)	15 (39.47%)	abi_milRNAs_13a<br>abi_milRNAs_23a<br>abi_milRNAs_18a

				abi_milRNAs_17a<br>abi_milRNAs_21a<br>abi_milRNAs_15a<br>abi_milRNAs_16a
Kalmanozyma brasiliensis	0 (0.00%)	2 (5.26%)	5 (13.16%)	abi_milRNAs_18a<br>abi_milRNAs_6a
Kwoniella bestiolae	0 (0.00%)	1 (2.63%)	2 (5.26%)	abi_milRNAs_16a
Kwoniella dejecticola	0 (0.00%)	2 (5.26%)	4 (10.53%)	abi_milRNAs_25a<br>abi_milRNAs_16a
Kwoniella heveanensis	0 (0.00%)	0 (0.00%)	1 (2.63%)	-----
Kwoniella mangroviensis	1 (2.63%)	1 (2.63%)	1 (2.63%)	abi_milRNAs_16a
Kwoniella pini	1 (2.63%)	1 (2.63%)	2 (5.26%)	abi_milRNAs_16a
Laccaria amethystina	9 (23.68%)	11 (28.95%)	16 (42.11%)	abi_milRNAs_13a<br>abi_milRNAs_27a<br>abi_milRNAs_8a<br>abi_milRNAs_23a<br>abi_milRNAs_9b<br>abi_milRNAs_14a<br>abi_milRNAs_19a<br>abi_milRNAs_21a<br>abi_milRNAs_15a<br>abi_milRNAs_9b
Laccaria bicolor	9 (23.68%)	14 (36.84%)	17 (44.74%)	abi_milRNAs_13a<br>abi_milRNAs_27a<br>abi_milRNAs_8a<br>abi_milRNAs_23a<br>abi_milRNAs_18a<br>abi_milRNAs_14a<br>abi_milRNAs_19a<br>abi_milRNAs_17a<br>abi_milRNAs_21a

				abi_milRNAs_15a<br>abi_milRNAs_16a<br>abi_milRNAs_9b<br>abi_milRNAs_6a
Laetiporus sulphureus	2 (5.26%)	5 (13.16%)	8 (21.05%)	abi_milRNAs_8a<br>abi_milRNAs_23a<br>abi_milRNAs_9b<br>abi_milRNAs_14a<br>abi_milRNAs_17a
Lentinula edodes	5 (13.16%)	9 (23.68%)	17 (44.74%)	abi_milRNAs_8a<br>abi_milRNAs_23a<br>abi_milRNAs_20a<br>abi_milRNAs_18a<br>abi_milRNAs_14a<br>abi_milRNAs_19a<br>abi_milRNAs_17a<br>abi_milRNAs_16a<br>abi_milRNAs_6a
Leucoagaricus spp	13 (34.21%)	16 (42.11%)	19 (50.00%)	abi_milRNAs_13a<br>abi_milRNAs_25a<br>abi_milRNAs_27a<br>abi_milRNAs_8a<br>abi_milRNAs_23a<br>abi_milRNAs_20a<br>abi_milRNAs_18a<br>abi_milRNAs_9b<br>abi_milRNAs_19a<br>abi_milRNAs_27b<br>abi_milRNAs_17a<br>abi_milRNAs_21a<br>abi_milRNAs_15a<br>abi_milRNAs_16a<br>abi_milRNAs_6a

Malassezia pachydermatis	0 (0.00%)	3 (7.89%)	5 (13.16%)	abi_milRNAs_18a<br>abi_milRNAs_16a<br>abi_milRNAs_6a
Malassezia sympodialis	0 (0.00%)	2 (5.26%)	2 (5.26%)	abi_milRNAs_18a<br>abi_milRNAs_6a
Mixia osmundae	0 (0.00%)	1 (2.63%)	3 (7.89%)	abi_milRNAs_18a
Moesziomyces antarcticus	0 (0.00%)	2 (5.26%)	4 (10.53%)	abi_milRNAs_18a<br>abi_milRNAs_6a
Moesziomyces aphidis	0 (0.00%)	2 (5.26%)	3 (7.89%)	abi_milRNAs_18a<br>abi_milRNAs_6a
Moniliophthora perniciosa	3 (7.89%)	8 (21.05%)	12 (31.58%)	abi_milRNAs_8a<br>abi_milRNAs_23a<br>abi_milRNAs_18a<br>abi_milRNAs_14a<br>abi_milRNAs_19a<br>abi_milRNAs_17a<br>abi_milRNAs_15a<br>abi_milRNAs_6a
Moniliophthora roreri	7 (18.42%)	10 (26.32%)	16 (42.11%)	abi_milRNAs_13a<br>abi_milRNAs_32a<br>abi_milRNAs_8a<br>abi_milRNAs_23a<br>abi_milRNAs_20a<br>abi_milRNAs_18a<br>abi_milRNAs_14a<br>abi_milRNAs_17a<br>abi_milRNAs_15a<br>abi_milRNAs_6a
Neolentinus lepideus	2 (5.26%)	3 (7.89%)	11 (28.95%)	abi_milRNAs_13a<br>abi_milRNAs_8a<br>abi_milRNAs_23a
Paxillus involutus	7 (18.42%)	10 (26.32%)	19 (50.00%)	abi_milRNAs_13a<br>abi_milRNAs_8a

				abi_milRNAs_23a<br>abi_milRNAs_18a<br>abi_milRNAs_19a<br>abi_milRNAs_17a<br>abi_milRNAs_21a<br>abi_milRNAs_15a<br>abi_milRNAs_16a
Paxillus rubicundulus	5 (13.16%)	7 (18.42%)	14 (36.84%)	abi_milRNAs_13a<br>abi_milRNAs_8a<br>abi_milRNAs_23a<br>abi_milRNAs_18a<br>abi_milRNAs_17a<br>abi_milRNAs_21a<br>abi_milRNAs_15a
Phanerochaete carnosa	6 (15.79%)	10 (26.32%)	17 (44.74%)	abi_milRNAs_8a<br>abi_milRNAs_23a<br>abi_milRNAs_18a<br>abi_milRNAs_14a<br>abi_milRNAs_19a<br>abi_milRNAs_17a<br>abi_milRNAs_21a<br>abi_milRNAs_15a<br>abi_milRNAs_16a<br>abi_milRNAs_6a
Phlebia centrifuga	8 (21.05%)	10 (26.32%)	15 (39.47%)	abi_milRNAs_8a<br>abi_milRNAs_23a<br>abi_milRNAs_18a<br>abi_milRNAs_14a<br>abi_milRNAs_19a<br>abi_milRNAs_17a<br>abi_milRNAs_21a

				abi_milRNAs_15a<br>abi_milRNAs_16a<br>abi_milRNAs_6a
Phlebiopsis gigantea	5 (13.16%)	10 (26.32%)	12 (31.58%)	abi_milRNAs_27a<br>abi_milRNAs_8a<br>abi_milRNAs_23a<br>abi_milRNAs_14a<br>abi_milRNAs_27b<br>abi_milRNAs_17a<br>abi_milRNAs_21a<br>abi_milRNAs_15a<br>abi_milRNAs_16a<br>abi_milRNAs_6a
Piloderma croceum	3 (7.89%)	3 (7.89%)	8 (21.05%)	abi_milRNAs_8a<br>abi_milRNAs_23a<br>abi_milRNAs_17a
Pisolithus microcarpus	6 (15.79%)	8 (21.05%)	12 (31.58%)	abi_milRNAs_13a<br>abi_milRNAs_25a<br>abi_milRNAs_8a<br>abi_milRNAs_23a<br>abi_milRNAs_17a<br>abi_milRNAs_21a<br>abi_milRNAs_15a<br>abi_milRNAs_6a
Pisolithus tinctorius	6 (15.79%)	10 (26.32%)	13 (34.21%)	abi_milRNAs_13a<br>abi_milRNAs_25a<br>abi_milRNAs_8a<br>abi_milRNAs_23a<br>abi_milRNAs_9b<br>abi_milRNAs_17a<br>abi_milRNAs_21a<br>abi_milRNAs_15a<br>abi_milRNAs_9b<br>abi_milRNAs_6a

Pleurotus ostreatus	8 (21.05%)	12 (31.58%)	16 (42.11%)	abi_milRNAs_13a<br>abi_milRNAs_8a<br>abi_milRNAs_23a<br>abi_milRNAs_20a<br>abi_milRNAs_18a<br>abi_milRNAs_9b<br>abi_milRNAs_19a<br>abi_milRNAs_17a<br>abi_milRNAs_21a<br>abi_milRNAs_16a<br>abi_milRNAs_9b<br>abi_milRNAs_6a
Plicaturopsis crispa	2 (5.26%)	7 (18.42%)	11 (28.95%)	abi_milRNAs_8a<br>abi_milRNAs_23a<br>abi_milRNAs_20a<br>abi_milRNAs_14a<br>abi_milRNAs_17a<br>abi_milRNAs_21a<br>abi_milRNAs_16a
Postia placenta	5 (13.16%)	10 (26.32%)	17 (44.74%)	abi_milRNAs_8a<br>abi_milRNAs_23a<br>abi_milRNAs_20a<br>abi_milRNAs_18a<br>abi_milRNAs_14a<br>abi_milRNAs_19a<br>abi_milRNAs_17a<br>abi_milRNAs_21a<br>abi_milRNAs_15a<br>abi_milRNAs_16a
Pseudozyma hubeiensis	0 (0.00%)	2 (5.26%)	4 (10.53%)	abi_milRNAs_18a<br>abi_milRNAs_6a
Puccinia sorghi	1 (2.63%)	4 (10.53%)	8 (21.05%)	abi_milRNAs_8a<br>abi_milRNAs_20a

				abi_milRNAs_18a<br>abi_milRNAs_6a
Puccinia striiformis	0 (0.00%)	1 (2.63%)	9 (23.68%)	abi_milRNAs_8a
Punctularia strigosozonata	6 (15.79%)	10 (26.32%)	15 (39.47%)	abi_milRNAs_13a<br>abi_milRNAs_8a<br>abi_milRNAs_23a<br>abi_milRNAs_18a<br>abi_milRNAs_14a<br>abi_milRNAs_17a<br>abi_milRNAs_21a<br>abi_milRNAs_15a<br>abi_milRNAs_16a<br>abi_milRNAs_6a
Rhizoctonia solani	1 (2.63%)	5 (13.16%)	10 (26.32%)	abi_milRNAs_25a<br>abi_milRNAs_18a<br>abi_milRNAs_19a<br>abi_milRNAs_17a<br>abi_milRNAs_16a
Rhizopogon vesiculosus	6 (15.79%)	11 (28.95%)	15 (39.47%)	abi_milRNAs_13a<br>abi_milRNAs_25a<br>abi_milRNAs_8a<br>abi_milRNAs_23a<br>abi_milRNAs_9b<br>abi_milRNAs_17a<br>abi_milRNAs_7a<br>abi_milRNAs_21a<br>abi_milRNAs_15a<br>abi_milRNAs_16a
Rhodotorula graminis	0 (0.00%)	1 (2.63%)	5 (13.16%)	abi_milRNAs_18a
Rhodotorula toruloides	0 (0.00%)	3 (7.89%)	4 (10.53%)	abi_milRNAs_25a<br>abi_milRNAs_18a<br>abi_milRNAs_6a

Schizophyllum commune	2 (5.26%)	4 (10.53%)	8 (21.05%)	abi_milRNAs_17a<br>abi_milRNAs_15a<br>abi_milRNAs_16a<br>abi_milRNAs_6a
Schizopora paradoxa	3 (7.89%)	7 (18.42%)	11 (28.95%)	abi_milRNAs_13a<br>abi_milRNAs_23a<br>abi_milRNAs_9b<br>abi_milRNAs_14a<br>abi_milRNAs_17a<br>abi_milRNAs_9b<br>abi_milRNAs_6a
Scleroderma citrinum	6 (15.79%)	9 (23.68%)	14 (36.84%)	abi_milRNAs_13a<br>abi_milRNAs_25a<br>abi_milRNAs_8a<br>abi_milRNAs_23a<br>abi_milRNAs_14a<br>abi_milRNAs_17a<br>abi_milRNAs_21a<br>abi_milRNAs_15a<br>abi_milRNAs_6a
Serendipita indica	0 (0.00%)	1 (2.63%)	6 (15.79%)	abi_milRNAs_14a
Serendipita vermifera	1 (2.63%)	2 (5.26%)	8 (21.05%)	abi_milRNAs_18a<br>abi_milRNAs_14a
Serpula lacrymans	5 (13.16%)	9 (23.68%)	18 (47.37%)	abi_milRNAs_13a<br>abi_milRNAs_8a<br>abi_milRNAs_23a<br>abi_milRNAs_20a<br>abi_milRNAs_18a<br>abi_milRNAs_19a<br>abi_milRNAs_17a<br>abi_milRNAs_15a<br>abi_milRNAs_16a

Sistotremastrum niveocremeum	2 (5.26%)	4 (10.53%)	10 (26.32%)	abi_milRNAs_13a<br>abi_milRNAs_23a<br>abi_milRNAs_19a<br>abi_milRNAs_6a
Sistotremastrum suecicum	2 (5.26%)	3 (7.89%)	7 (18.42%)	abi_milRNAs_13a<br>abi_milRNAs_23a<br>abi_milRNAs_6a
Sphaerobolus stellatus	0 (0.00%)	2 (5.26%)	10 (26.32%)	abi_milRNAs_32a<br>abi_milRNAs_17a
Sporidiobolus salmonicolor	0 (0.00%)	2 (5.26%)	3 (7.89%)	abi_milRNAs_19a<br>abi_milRNAs_6a
Sporisorium scitamineum	0 (0.00%)	2 (5.26%)	5 (13.16%)	abi_milRNAs_18a<br>abi_milRNAs_6a
Stereum hirsutum	4 (10.53%)	7 (18.42%)	15 (39.47%)	abi_milRNAs_13a<br>abi_milRNAs_8a<br>abi_milRNAs_23a<br>abi_milRNAs_18a<br>abi_milRNAs_17a<br>abi_milRNAs_21a<br>abi_milRNAs_6a
Suillus luteus	5 (13.16%)	6 (15.79%)	12 (31.58%)	abi_milRNAs_13a<br>abi_milRNAs_8a<br>abi_milRNAs_23a<br>abi_milRNAs_17a<br>abi_milRNAs_21a<br>abi_milRNAs_15a
Termitomyces spp	5 (13.16%)	11 (28.95%)	15 (39.47%)	abi_milRNAs_13a<br>abi_milRNAs_8a<br>abi_milRNAs_23a<br>abi_milRNAs_9b<br>abi_milRNAs_14a<br>abi_milRNAs_17a

				abi_milRNAs_21a<br>abi_milRNAs_15a<br>abi_milRNAs_16a<br>abi_milRNAs_6a
Tilletia caries	0 (0.00%)	1 (2.63%)	3 (7.89%)	abi_milRNAs_18a
Tilletia controversa	0 (0.00%)	1 (2.63%)	3 (7.89%)	abi_milRNAs_18a
Tilletiaria anomala	0 (0.00%)	0 (0.00%)	2 (5.26%)	-----
Trametes cinnabarina	8 (21.05%)	13 (34.21%)	14 (36.84%)	abi_milRNAs_8a<br>abi_milRNAs_23a<br>abi_milRNAs_20a<br>abi_milRNAs_18a<br>abi_milRNAs_9b<br>abi_milRNAs_14a<br>abi_milRNAs_19a<br>abi_milRNAs_17a<br>abi_milRNAs_21a<br>abi_milRNAs_15a<br>abi_milRNAs_16a<br>abi_milRNAs_6a
Trametes pubescens	8 (21.05%)	13 (34.21%)	16 (42.11%)	abi_milRNAs_8a<br>abi_milRNAs_23a<br>abi_milRNAs_20a<br>abi_milRNAs_18a<br>abi_milRNAs_9b<br>abi_milRNAs_14a<br>abi_milRNAs_19a<br>abi_milRNAs_17a<br>abi_milRNAs_21a<br>abi_milRNAs_15a<br>abi_milRNAs_16a<br>abi_milRNAs_9b<br>abi_milRNAs_6a
Trametes versicolor	7 (18.42%)	11 (28.95%)	12 (31.58%)	abi_milRNAs_8a

				abi_milRNAs_23a<br>abi_milRNAs_20a<br>abi_milRNAs_18a<br>abi_milRNAs_9b<br>abi_milRNAs_14a<br>abi_milRNAs_17a<br>abi_milRNAs_21a<br>abi_milRNAs_15a<br>abi_milRNAs_9b<br>abi_milRNAs_6a
Tremella mesenterica	1 (2.63%)	1 (2.63%)	3 (7.89%)	abi_milRNAs_18a
Trichosporon asahii	0 (0.00%)	2 (5.26%)	5 (13.16%)	abi_milRNAs_18a<br>abi_milRNAs_6a
Tsuchiyaea wingfieldii	0 (0.00%)	0 (0.00%)	3 (7.89%)	
Tulasnella calospora	0 (0.00%)	4 (10.53%)	9 (23.68%)	abi_milRNAs_23a<br>abi_milRNAs_14a<br>abi_milRNAs_17a<br>abi_milRNAs_6a
Ustilago hordei	0 (0.00%)	1 (2.63%)	2 (5.26%)	abi_milRNAs_6a
Wallemia ichthyophaga	0 (0.00%)	0 (0.00%)	7 (18.42%)	-----
Wallemia mellicola	0 (0.00%)	2 (5.26%)	5 (13.16%)	abi_milRNAs_32a<br>abi_milRNAs_18a
Xanthophyllomyces dendrorhous	1 (2.63%)	4 (10.53%)	7 (18.42%)	abi_milRNAs_20a<br>abi_milRNAs_18a<br>abi_milRNAs_19a<br>abi_milRNAs_16a

Table S4. List of Basidiomycetes species downloaded for the study of homology. The species are listed following taxonomical criteria. Source: Ensembl Fungi. (ftp://ftp.ensemblgenomes.org/pub/fungi/release-37/fasta/fungi_basidiomycota1_collection/).

**Cl. *Agaricomycetes,***	
O. *Agaricales*	
F. Agaricaceae:	Agaricus bisporus,
Leucoagaricus sp	
F. Amanitaceae:	Amanita muscaria
F. Amylocorticiaceae:	Plicaturopsis crispa

F. Fistulinacea:	Fistulina hepatica
F. Hymenogastraceae:	Hebeloma cylindrosporum
F. Lyophyllaceae:	Termitomyces sp
F. Marasmiaceae:	Lentinula edodes,
Moniliophthora perniciosa,	
Moniliophthora roreri	
F. Omphalotaceae:	Gymnopus luxurians
F. Pleurotaceae:	Pleurotus ostreatus
F. Schizophyllaceae:	Schizophyllum commune
F. Strophariaceae:	Galerina marginata,
Hypholoma sublateritium	
F. Tricholomataceae:	Hypsizygus marmoreus
O. Atheliales	
F. Atheliaceae:	Fibulorhizoctonia sp,
Piloderma croceum	
O. Auriculariales	
F. Auriculariaceae:	Exidia glandulosa
F. Physalacriaceae:	Cylindrobasidium torrendii
F. Psathyrellaceae:	Coprinopsis cinerea
O. Boletales	
F. Coniophoraceae:	Coniophora puteana
F. Paxillaceae:	Hydnomerulius pinastri,
Paxillus involutus,	
Paxillus rubicundulus	
F. Rhizopogonaceae:	Rhizopogon vesiculosus
F. Sclerodermataceae:	Pisolithus microcarpus,
Pisolithus tinctorius,	
Scleroderma citrinum	
F. Serpulceae:	Serpula lacrymans
F. Suillaceae:	Suillus luteus
O. Cantharellales	
F. Botryobasidiaceae:	Botryobasidium botryosum
F. Ceratobasidiaceae:	Rhizoctonia solani
F. Tiullasnellaceae:	Tulasnella calospora
O. Corticales	
F. Punctulariaceae:	Punctularia strigosozonata
O. Geastrales	
F. Sphaerobolaceae:	Sphaerobolus stellatus
O. Gloeophyllales	
F. Gloeophyllaceae:	Gloeophyllum trabeum,
Neolentinus lepideus	
O. Hymenochaetales	
F. Hymenochaetaceae:	Fomitiporia mediterranea,
Fomitopsis pinicola	
F. Hydnangiaceae:	Laccaria amethystina,
Laccaria bicolor	
F. Schizoporaceae:	Schizopora paradoxa
O. Jaapiales	
F. Jaapiaceae:	Jaapia argillacea
O. Polyporales	
F. Fomitopsidaceae:	Daedalea quercina,
Fibroporia radiculosa,	
Postia placenta	
F. Ganodermataceae:	Ganoderma lucidum
F. Gelatoporiaceae:	Gelatoporia subvermispora
F. Meripilaceae:	Grifola frondosa
F. Meruliaceae:	Phlebia centrifuga

F. Phanerochaetaceae:	Phanerochaete carnosa,
Phlebiopsis gigantea	
F. Polyporaceae:	Dichomitus squalens,
Laetiporus sulphureus,	
Trametes cinnabarina,	
Trametes pubescens	
	Trametes versicolor
O. Russulales:	
F. Bondarzewiaceae,	Heterobasidion irregulare
F. Steraceae:	Stereum hirsutum
O. Sebacinales	
F. Serendipitaceae:	Serendipita indica,
Serendipita vermifera	
O. Trechisporales	
F. Hydnodontaceae:	Sistotremastrum niveocremeum,
Sistotremastrum suecicum	
Cl. Dacrymycetes,	
O. Dacrymycetales	
F. Dacrymycetaceae:	Calocera cornea,
Calocera viscosa,	
Dacryopinax primogenitus	
Cl. Exobasidiomycetes,	
O. Ceraceosorales	
F. Ceraceosoraceae:	Ceraceosorus bombacis
O. Malasseziales	
F.Malasseziaceae:	Malassezia pachydermatis,
Malassezia sympodialis	
O. Tilletiales	
F. Tilletiaceae:	Tilletia caries,
Tilletia controversa	
O. Georgefischeriales	
F. Tilletiariaceae:	Tilletiaria anomala
Cl. Microbotryomycetes,	
O. Sporidiobolales	
F. Sporidiobolaceae:	Rhodotorula graminis, Rhodotorula toruloides, Sporidiobolus salmonicolor
Cl. Mixiomycetes,	
O. Mixiales	
F. Mixiaceae:	Mixia osmundae
Cl. Pucciniomycetes,	
O. Pucciniales	
F. Pucciniaceae:	Puccinia sorghi, Puccinia striiformis
Cl. Tremellomycetes,	
O. Cystofilobasidiales	
F. Cystofilobasidiaceae:	Xanthophyllomyces dendrorhous
O. Tremellales	
F. Cryptococcaceae:	Kwoniella bestiolae,
Kwoniella dejecticola,	
Kwoniella heveanensis,	
Kwoniella mangroviensis,	
Kwoniella pini	

F. Tremellaceae: Cryptococcus amylolentus,
Cryptococcus depauperatus,
Cryptococcus gattii,
Cryptococcus neoformans,
Tremella mesenterica,
Tsuchiyaea wingfieldii
O. Trichosporonales
F Trichosporonaceae: Cutaneotrichosporon oleaginosus,
Trichosporon asahii
Cl. Ustilaginomycetes,
O. Ustilaginales
F. Ustilaginaceae: Anthracocystis flocculosa,
Kalmanozyma brasiliensis,
Moesziomyces antarcticus, Moesziomyces aphidis,
Pseudozyma hubeiensis,
Sporisorium scitamineum,
Ustilago hordei
Cl. Wallemiomycete,
O. Wallemiales
F. Wallemiaceae: Wallemia ichthyophaga,
Wallemia mellicola

Table S5. Primers used for RT-qPCR experiments in this study. CMS: consensus mature sequence; CSS: consensus star sequence; CPS: consensus precursor sequence; HK: Housekeeping; Fw: Forward; Rev: Reverse.

ID	Primer sequence (5' → 3')	Length (nt)
abi_milRNAs_1a_CMS	GTGGGCTGGGCTGCTGCAGCG	21
abi_milRNAs_1a_CSS	GCTGTGGTGGCTTGTCCACG	20
abi_milRNAs_1a_CPS_Fw	CCGCACATGTGACTTGAGTT	20
abi_milRNAs_1a_CPS_Rev	TAACCTCAGCGGATCGTAGC	20
abi_milRNAs_2a_CMS	TCTAATCATGGACGTGCT	18
abi_milRNAs_2a_CSS	TAGCTCAGTGGTAGAGCGTG	20
abi_milRNAs_2a_CPS_Fw	CGAACGAACTATGCACTTGG	20
abi_milRNAs_2a_CPS_Rev	TGACCATCCGCACTAGAAAG	20
abi_milRNAs_4a_CMS	AGGCTGCGGAACGTTGGCACGGGT	24
abi_milRNAs_4a_CSS	TACTGTCGCCATTGGAAAACAGCTTTCT	28
abi_milRNAs_4a_CPS_Fw	CAGTACTGTCGCCATTGGAA	20
abi_milRNAs_4a_CPS_Rev	AATACGAGAGGCAGCTTGGA	20
rRNA_5.8S_HK_Fw	TGATTACGTCCCTGCCCTTT	20
rRNA_5.8S_HK_Rev	AAGAGCCAGCTACAACCCAT	20
